# Electrospun Polyvinylidene Fluoride-Based Fibrous Scaffolds with Piezoelectric Characteristics for Bone and Neural Tissue Engineering

**DOI:** 10.3390/nano9070952

**Published:** 2019-06-30

**Authors:** Yuchao Li, Chengzhu Liao, Sie Chin Tjong

**Affiliations:** 1 Department of Materials Science and Engineering, Liaocheng University, Liaocheng 252000, China; 2 Department of Materials Science and Engineering, Southern University of Science and Technology, Shenzhen 518055, China; 3 Department of Physics, City University of Hong Kong, Tat Chee Avenue, Kowloon, Hong Kong, China

**Keywords:** piezoelectricity, scaffold, polyvinylidene fluoride, polyvinylidene fluoride-trifluoroethylene, tissue engineering, osteoblast, neuron, stem cell, electrospinning, aligned fiber

## Abstract

Polyvinylidene fluoride (PVDF) and polyvinylidene fluoride-trifluoroethylene (P(VDF-TrFE) with excellent piezoelectricity and good biocompatibility are attractive materials for making functional scaffolds for bone and neural tissue engineering applications. Electrospun PVDF and P(VDF-TrFE) scaffolds can produce electrical charges during mechanical deformation, which can provide necessary stimulation for repairing bone defects and damaged nerve cells. As such, these fibrous mats promote the adhesion, proliferation and differentiation of bone and neural cells on their surfaces. Furthermore, aligned PVDF and P(VDF-TrFE) fibrous mats can enhance neurite growth along the fiber orientation direction. These beneficial effects derive from the formation of electroactive, polar β-phase having piezoelectric properties. Polar β-phase can be induced in the PVDF fibers as a result of the polymer jet stretching and electrical poling during electrospinning. Moreover, the incorporation of TrFE monomer into PVDF can stabilize the β-phase without mechanical stretching or electrical poling. The main drawbacks of electrospinning process for making piezoelectric PVDF-based scaffolds are their small pore sizes and the use of highly toxic organic solvents. The small pore sizes prevent the infiltration of bone and neuronal cells into the scaffolds, leading to the formation of a single cell layer on the scaffold surfaces. Accordingly, modified electrospinning methods such as melt-electrospinning and near-field electrospinning have been explored by the researchers to tackle this issue. This article reviews recent development strategies, achievements and major challenges of electrospun PVDF and P(VDF-TrFE) scaffolds for tissue engineering applications.

## 1. Introduction

The design of novel biomaterials for applications in hard tissue such as bone and soft tissue like nerves presents a big challenge for chemists, materials scientists and biomedical engineers. Advanced functional scaffolds for hard and soft tissue engineering applications should possess certain requirements including biocompatible, hydrophilic, porous and electro-mechanical characteristics. Piezoelectric materials can generate electrical charges in response to an applied stress or minute mechanical deformation, thus eliminating the need for external power sources for electrical stimulation. Typical piezoelectric materials include barium titanate, lead titanate and lead zirconate titanate ceramics as well electroactive polymers such as polyvinylidene fluoride (PVDF) [1,2,3,4,5]. However, the toxicity and environmental impacts of lead-based piezoceramics preclude their use for biomedical applications. In this respect, piezoelectric polymers exhibit several distinct advantages over piezoceramics including nontoxic, excellent flexibility, light weight and ease of fabrication. Therefore, PVDF and polyvinylidene fluoride-trifluoroethylene (P(VDF-TrFE) copolymer with excellent piezoelectricity and good processability can be tailored to form smart scaffolds to stimulate cell growth for tissue engineering applications [2,3,4,6]. By applying mechanical stresses to piezoelectric scaffolds, electrical stimulation is activated, and is transmitted to the neighboring cells, thereby enhancing cell signaling pathways for tissue regeneration [7,8]. Consequently, the biological electric field in the host tissues plays important roles in cellular membrane function, tissue growth and regeneration [9]. Electrospun PVDF fibrous mats that mimic the structure and electrical response of natural extracellular matrix (ECM) are considered of clinical importance. This is because electrospun PVDF scaffolds can promote bone generation, osteogenic and neural stem cell differentiation [10,11,12,13,14,15,16,17]. Human bones generally exhibit piezoelectric properties [18,19]. Bones consist of collagen fibrils and nanohydroxyapatite (nHA) that control biomechanical motion of humans [20]. The properties of collagen are dictated by the peptide bond. The carbonyl oxygen of peptide has a negative charge, while the amide nitrogen with a positive charge, thus establishing a small electric dipole [21]. When collagen is mechanically deformed, electric charges are produced, and the electrical potential generated promotes bone growth and regeneration [22,23,24,25,26].

Recently, bone defects and diseases remain a major health issue globally because of the rise in injuries due to osteoporosis, bone cancer, sport activity and traffic accident [27,28,29,30,31,32]. This leads to an urgent need in the biomedical sector to develop biocompatible substitutes to replace and regenerate defective bone tissues [33]. Moreover, neurotrauma due to traumatic brain injury and neurodegenerative diseases, such as Alzheimer’s, Parkinson’s, and stroke, are becoming increasingly prevalent among elderly patients [34,35,36]. Degenerative diseases are caused by a progressive loss of function of nerve cells of human nervous system. Regeneration of the nervous system requires the repair or replacement of nerve cells that have been damaged by injury or disease. However, human central nervous system has limited capability to regenerate damaged nerve cells [37]. As such, stem cell therapy is a promising route to treat neurological disorders because stem cells can differentiate into multiple cell types including neurons, thus serving as a source for cell replacement of damaged cells [38]. In this respect, tissue engineering and regenerative medicine provide potential solutions for creating novel smart scaffolds for self-repairing, remodeling and regeneration of bone and nerve tissues [39,40,41,42].

**Figure 1:**
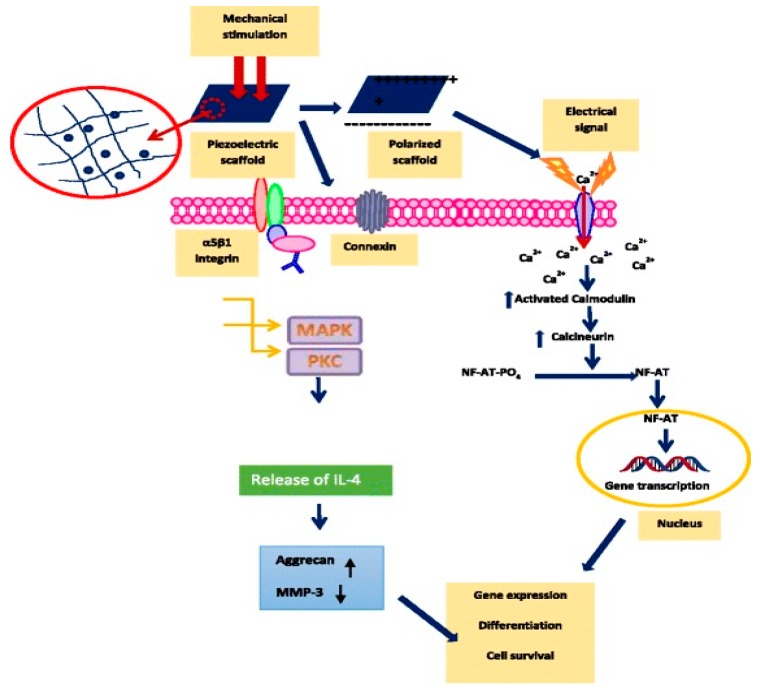
Schematic diagram showing the activation of Ca^2+^ signal transduction pathway and other miscellaneous pathways in response to the electrical and mechanical stimulations. Ca^2+^ is an important signal transducer.

As is widely recognized, electrical charge stimulation greatly influences cellular behavior by affecting ion channel function across the cell membrane, monitoring the membrane potential and regulating the intracellular signal transduction pathways. Therefore, the cells can respond directionally to an applied electric field in vitro and in vivo [43]. Conductive polymers such as polypyrrole, polyaniline, poly(3,4-ethylenedioxythiophene), polythiophene, etc., have been shown to enhance and direct neurite outgrowth on their surfaces [44]. However, the application of those polymers has been hampered in the clinical sector due to the requirement of wired electrical stimulation with external power sources, thus increasing the risk of infection and inflammation [45]. Accordingly, electroactive PVDF and its copolymers, which can generate electrical charges on its surface upon mechanical or electrical stimulation, are very attractive for tissue engineering applications [3,46]. The generated charges and electrical dipoles would stimulate bone remodeling and growth through the opening of voltage-gated calcium ion channels [47]. Thus, the calcium/calmodulin pathway of bone cells is activated, facilitating osteogenic differentiation and proliferation (Figure 1) [8]. Tissue engineering is a multidisciplinary field combining cell biology, biochemistry, materials science, medicine and engineering disciplines to restore, maintain and repair damaged tissues and organs. 

PVDF exhibits excellent electroactive behaviors, good biocompatibility, excellent chemical resistance, and thermal stability, rendering it an attractive material for biomedical, electronic, environmental and energy harvesting applications [3,4,48,49,50,51,52]. For instance, PVDF with good biocompatibility and processability can be used to make monofilament suture material for cardiovascular surgery [52]. PVDF is a semicrystalline polymer having five crystalline polymorphs, including α-, β-, γ- δ- and ε-phases [2,53,54,55]. Among these, the β-PVDF-phase exhibits excellent piezoelectric and ferroelecric properties. The β-phase can be promoted in PVDF by either mechanical drawing, annealing, electrical poling or nanoparticle additions [54,55,56,57,58,59,60]. Aided by recent advances in nanotechnology, a wide range of nanomaterials can be synthesized for biomedical and industrial applications [61,62,63,64,65,66,67,68,69,70,71,72]. In particular, clay nanoplatelets, carbon nanotubes, graphene/graphene oxide and silica nanoparticles have been reported to be very effective to induce β-phase in PVDF [57,59,73,74,75,76,77,78,79,80,81,82]. As is generally recognized, electrospinning can fabricate micro- and nanofibers with interconnecting pores, resembling the natural ECM in tissues [83]. Thus, it is an effective technique for preparing smart PVDF fibrous scaffolds with piezoelectric characteristics. Sundaray et al. indicated that electrospinning could induce a change of crystalline structure of PVDF from non-polar α-phase to polar β-phase. This was ascribed to the intense stretching of the PVDF jet during the electrospinning process [84]. The β-phase content can be enhanced by monitoring electrospinning parameters [85]. This article provides the state-of-the art review on the development and piezoelectric properties of electrospun PVDF and P(VDF-TrFE) scaffolds for tissue engineering applications.

## 2. Structural Behavior

**Figure 2:**
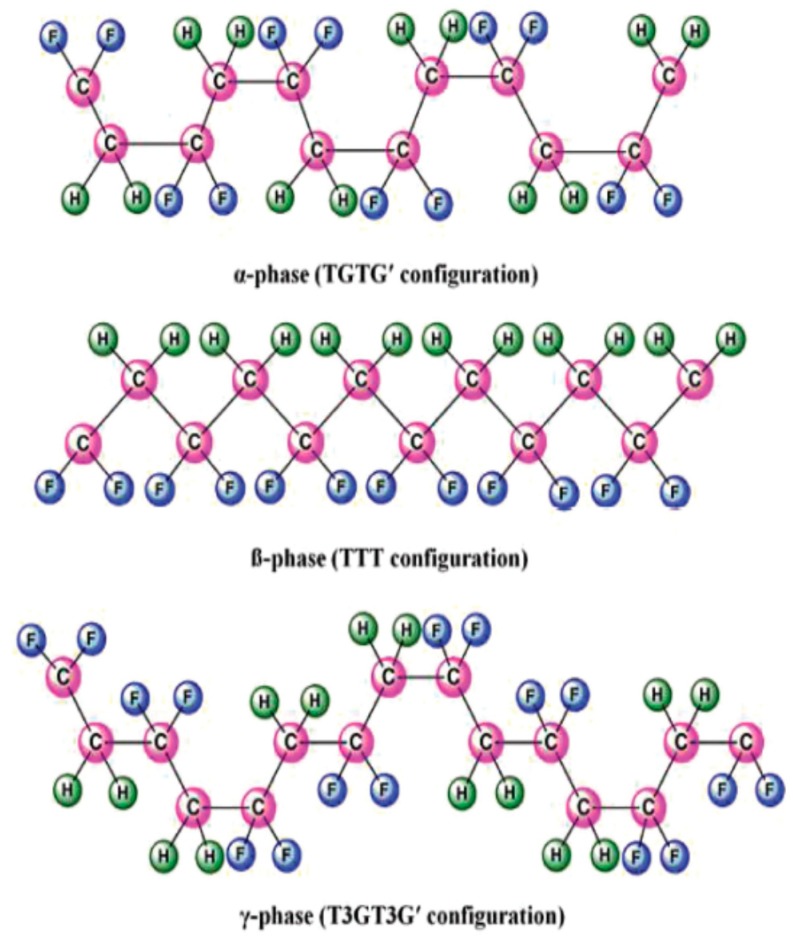
Primary polymorphic crystalline phases of PVDF. Reproduced with permission from [50], published by Wiley-VCH, 2019.

**Figure 3:**
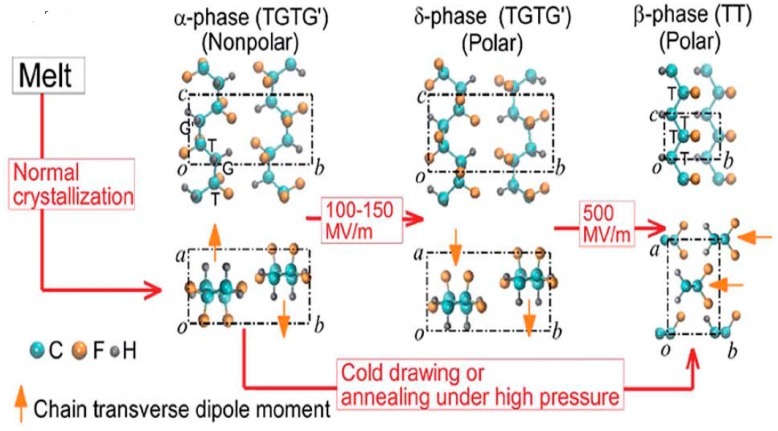
Electric field-induced phase transitions of PVDF. Electric poling aligns the dipoles along the electric field by applying a very high voltage. The transverse dipole moment of each polymer chain is shown using an orange arrow that points from the negatively charged fluorine atoms to the positively charged hydrogen atoms. T-trans; G-gauche. Reproduced with permission from [55], published by AIP Publishing, 2016.

PVDF, with the chemical formula (CH_2_–CF_2_)n, possesses good piezoelectricity. Its molecular chain consists of highly electronegative fluorine atoms compared to the carbon and hydrogen atoms. This leads to the formation of polar C–F bonds, and each C–F bond possesses a significant dipole moment. PVDF exhibits five crystalline polymorphs including nonpolar α- and ε-phases, and polar β-, γ- and δ-phases depending on the crystallization and processing conditions [2,48,49,50]. Figure 2 shows a typical chain conformation for α, β and γ crystalline phases of PVDF [50]. The most common polymorph is α-PVDF that crystallized readily from the melt [51]. Nonpolar α-PVDF is monoclinic with its unit cell containing two chains in an alternating trans-gauche-trans-gauche′ (TGTG′) conformation. As a result, the net dipole moment cancels out due to its antiparallel molecular chain arrangement (Figure 3). The α-PVDF can be transformed into three other polymorphic forms under the application of mechanical stress (cold drawing), electrical field or annealing treatment. The δ-phase exhibits the same TGTG′ conformation of macromolecular chains, but all dipoles are arranged parallel to each other, resulting in ferroelectric behavior. This phase can be produced by poling α-PVDF at an applied electric field of 100–150 MV/m. The hydrogen and fluorine atoms flip with respect to the carbon backbone. However, this process often leads to the breakdown of both the electrode and the polymer [86]. Moreover, δ-phase can transform to β-phase, with the hydrogen, fluorine, and carbon atoms all moving to produce the all-trans configuration under a high electric field (~500 MV/m) (Figure 3) [55]. The β-PVDF phase is orthorhombic with all-trans (TTTT) planar zigzag conformation, having all dipoles aligned in the same direction normal to the chain axis. So β-PVDF phase can generate the highest spontaneous polarization, showing strong ferroelectric and piezoelectric properties. It is unlikely to form β-phase from the melt, because of the high energy barrier of the all-trans conformation. The α-phase can transform to the β-phase under mechanical drawing, annealing treatment at high pressure and electrical poling (Figure 3). The γ-phase also has an orthorhombic unit cell, and is characterized by a sequence of trans and gauche conformation (T3GT3G′). This phase can be obtained by high-temperature drawing of ultrahigh molecular weight PVDF [87].

PVDF can be copolymerized with TrFE, i.e.,–(CHF–CF_2_)–, and tetrafluoroethylene (TeFE) [–(CF_2_–CF_2_)–], in a random sequence. P(VDF-TrFE) crystallizes readily from the melt and forms the β-phase through copolymerization without mechanical stretching or drawing. This is due to the extra fluorine atoms introduced into the molecular chain causing a steric hindrance effect, thus preventing the formation of α-phase. The co-monomer extends the interchain distance and reduces the activation energy for the α-phase to β-phase. Additional annealing, mechanical stretching or electrical poling treatments can lead to a further increase in the degree of crystallinity and alignment of the CF_2_ dipoles, thereby producing higher piezo- and pyro-electric effects than PVDF [54]. P(VDF-TrFE) generally exhibits all trans conformation having TrFE content ranging 20–50 mol%. At TrFE content <20 mol%, P(VDF-TrFE) possesses mixed phases of α, β, and γ [88,89,90]. Thus, the structural, ferroelectric and piezoelectric properties of P(VDF-TrFE) depend greatly on the TrFE content [91].

**Figure 4:**
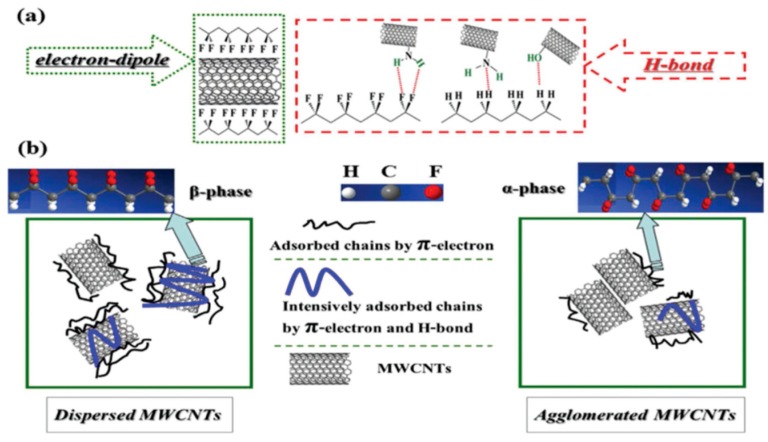
Schematic showing the effect of MWCNTs on the β-phase formation in PVDF: (**a**) Hydrogen bonding between functionalized MWCNTs and PVDF chains; (**b**) the adsorbed chains of PVDF on the surface of MWCNTs influenced by the dispersion of MWCNTs. Reproduced with permission from [96], published by Elsevier, 2014.

As mentioned above, nanoparticle additions can promote formation of the β-phase in PVDF by acting as effective nucleating agents. Nanofillers with high surface-to-volume ratios can enhance their interactions with the polymer matrices. These nanomaterials include carbon nanofiber, carbon nanotube, graphene oxide (GO), and barium titanate [57,58,59,60,74,75,76,77,78,79,80,81]. Furthermore, metal (e.g., silver) and metal oxide nanoparticles like zinc oxide, and titania can also induce β-PVDF formation [82,92,93,94,95]. The extent of β-phase induced depends greatly on the properties of nanomaterials (e.g., type, shape, and size), surface chemistry and dispersion state. For instance, Ke et al. functionalized multiwalled carbon nanotubes (MWCNTs) with amino, carboxyl and hydroxyl groups, and then melt compounded with PVDF. They reported that amino group-functionalized MWCNTs (NH_2_–MWCNTs) induced the highest β-phase content (17.4%), followed by those with hydroxyl groups (11.6%) and unmodified MWCNTs (9.4%). The nanocomposites with carboxyl-functionalized nanotubes exhibited the lowest β-phase content (4.7%) (Figure 4) [96]. The interfacial interactions between the amino or hydroxyl groups of MWCNTs with the CH_2_/CF_2_ dipoles of PVDF facilitates the formation of hydrogen bonding. Moreover, the combined effects of the MWCNTs dispersion and the nanotube–PVDF interaction were responsible for the formation of β-phase in PVDF (Figure 4). El Achaby et al. prepared PVDF/GO nanocomposites by means of solvent casting [77]. They reported that the strong interfacial interactions between carbonyl group of GO and fluorine group (CF_2_) of PVDF led to the homogeneous dispersion of GO sheets in the PVDF matrix. Consequently, a small amount of GO (0.1 wt%) was needed to induce electroactive β-phase. 

**Figure 5:**
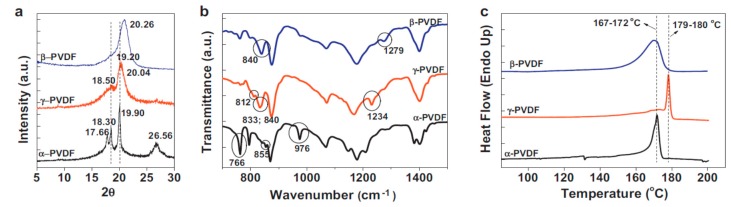
(**a**) XRD patterns, (**b**) FTIR spectra, and (**c**) DSC curves of the polymorphs of PVDF. Reproduced with permission from [2], published by Elsevier, 2014.

The polymorphs of PVDF and its copolymers are typically identified by means of X-ray diffraction (XRD), Fourier transform infrared spectroscopy (FTIR), and differential scanning calorimetry (DSC), as shown in Figure 5 [2]. The XRD pattern of α-PVDF shows the presence of diffraction peaks at 17.7°, 18.3°, 19.9° and 26.5°, corresponding to the (100), (020), (110), and (021) reflections. The XRD pattern of β-PVDF displays a characteristic peak at 20.26°, corresponding to the diffracting planes of (110) and (200). The γ-PVDF exhibits characteristic peaks at 18.5°, 19.2° and 20.04°, assigned to the (020), (002) and (110) reflections, respectively (Figure 5a) [2]. FTIR is particularly useful for identifying vibrational modes of the molecular chains of PVDF with different polymorphs, and for quantifying the amount of β-phase content. From Figure 5b, the vibrational bands of α-PVDF are located at 530 cm^−1^ (CF_2_ bending), 615 cm^−1^ (CF_2_ bending and skeletal bending), 766 cm^−1^ (CF_2_ bending and skeletal bending), 795 cm^−1^ (CH_2_ rocking), 855 cm^−1^ (CH out-of-plane deformation) and 976 cm^−1^ (CH out-of-plane deformation). The bands of β-PVDF appear at 510 cm^−1^ (CF_2_ bending), 840 cm^−1^ (CH_2_ rocking) and 1279 cm^−1^ (CF out-of-plane deformation). The characteristic bands of γ-PVDF are located at 776 cm^−1^ (CH_2_ rocking), 812 cm^−1^ (CH_2_ out-of-plane wag), 833 cm^−1^, 840 cm^−1^ (CH_2_ rocking), and 1234 cm^−1^ (CF out-of-plane deformation) [97]. Furthermore, the fraction of β-phase, F(β), can be quantitatively determined from the following equation [98],

F(β) = A_β_/[(K_β_/K_α_)A_α_ + A_β_] (1)

where A_α_ and A_β_ are the absorbance of α- and β-phases at 766 and 840 cm^−1^, and K_α_ (6.1 × 10^4^ cm^2^/mol) and K_β_ (7.7 × 10^4^ cm^2^/mol) are the absorption coefficients at the respective wavenumbers [2]. DSC is a powerful tool to characterize the melting and crystallization behaviors of PVDF [99]. The melting temperature (Figure 5c), crystallization temperature and the degree of crystallinity can be obtained from the DSC scans. All these parameters depend greatly on the structural conformation and molecular weight of PVDF. 

**Figure 6:**
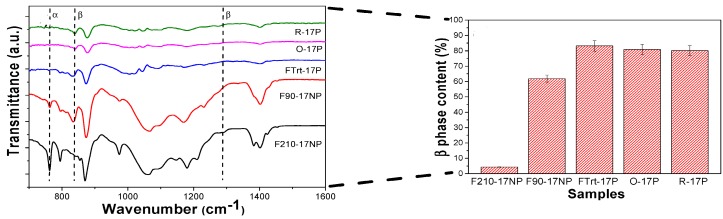
FTIR spectra of nonporous PVDF/silica (17 nm) nanocomposite films processed at 90 °C (F90-17NP) and at 210 °C (F210-17NP), porous nanocomposite film processed at room temperature (FTrt-17P), electrospun nanocomposite mats with oriented (O-17P) and random (R-17P) fibers. Right panel displays the β-phase content of these nanocomposites.

Very recently, Lanceros-Méndez and coworkers prepared PVDF/silica (17 nm) nanocomposites at different processing temperatures. They also fabricated porous nanocomposite mats using electrospinning [100]. The β-phase contents of electrospun nanocomposite mats with oriented (O-17P) and random (R-17P) fibers determined from Equation (1) were about 80% and 79.5%, respectively (Figure 6). A similarly high β-phase content was observed in porous nanocomposite film processed at room temperature (FTrt-17P). The high β-phase contents in porous electrospun nanocomposite mats derived from the solvent evaporation at room temperature and macromolecular chain stretching during the fiber formation. However, nonporous composite film prepared at 210 °C (F210-17NP) through a melting and recrystallization had a very low β-phase content; this film crystallized mainly into the α-phase. It appeared that silica nanoparticles had little influence on inducing the β-phase. Thus, processing temperature and electrospinning conditions are the main factors affecting the β-phase content in those nanocomposites [100].

## 3. Scaffold Fabrication

**Figure 7:**
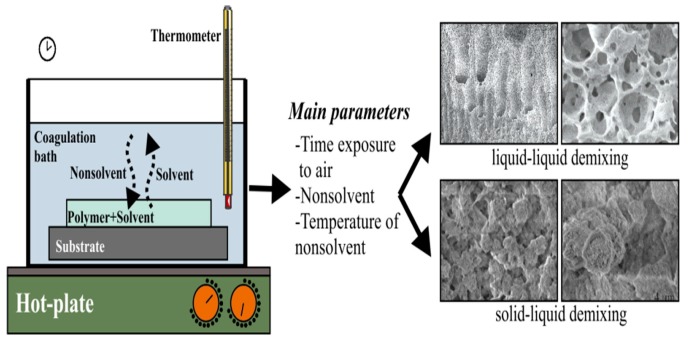
Porous scaffolds produced by non-solvent induced phase separation (NIPS). Reproduced with permission from [112], published by Elsevier, 2015.

The scaffolds for tissue engineering applications should be bioactive and biocompatible with sufficient porosity, pore size and highly interconnected pore network for the repair and regeneration of tissues. They should also possess high mechanical strength for supporting cell adhesion, growth and proliferation, and for transporting nutrient and metabolic waste [40,101,102,103]. PVDF with good processability, flexibility and low-cost can be readily fabricated into porous scaffolds for tissue engineering applications. Functional scaffolds based on PVDF and its copolymers can be prepared using conventional processing techniques, including solvent-casting/particulate leaching, solvent-casting and 3D polymer template, non-solvent induced phase separation (NIPS), and thermally induced phase separation (TIPS) [3,4,104,105,106,107,108]. The solvent casting/particulate leaching is a relatively simple process for forming scaffolds with a high porosity (up to 93%) and pore sizes of up to 500 µm [109]. In this process, a polymer is first dissolved in an organic solvent, mixed with porogens such as salt or sugar, and then cast into a mold. Subsequently, the mold is dipped in a water bath to dissolve the porogens. The pore volume, pore size and pore shape are governed by the amount, size and shape of porogens added. The main drawbacks of this technique include a wide variation of the pore sizes, poor pore interconnectivity, and irregular pore geometry [110]. Accordingly, several approaches have been developed to address some of these issues. For instance, combination of solvent casting and polymer templates (e.g., polyvinyl alcohol or nylon) can yield PVDF scaffolds with interconnected and well-distributed pores [104,105]. Alternatively, a phase inversion method can be used to fabricate microporous scaffolds with interconnected pores. Phase inversion occurs when a change takes place in the stability of a polymer solution as a result of the temperature variation, solvent evaporation, or mass exchange with nonsolvent [111,112]. In the NIPS or immersion precipitation approach, the polymer/solvent solution is immersed in a coagulation bath containing nonsolvent (e.g., water). This leads to a mass transfer between the solvent and nonsolvent, i.e., the solvent diffuses to the nonsolvent, while nonsolvent penetrates into the polymer solution (Figure 7). Consequently, the polymer solution becomes thermodynamically unstable, resulting in the phase separation, either by liquid-liquid or solid liquid demixing. This causes precipitation of a polymer-rich phase and a polymer lean-phase [113]. Upon the removal of the solvent, polymer-rich phase develops into a continuous matrix of the scaffold, while the polymer lean-phase forms the porous tunnels within the matrix, resulting in an interconnected porous network.

**Figure 8:**
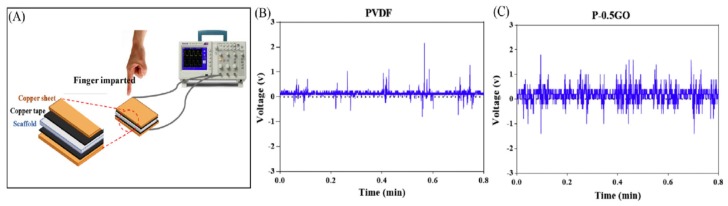
(**A**) Schematic of piezoelectricity measurement. Output voltage generation from representative (**B**) PVDF and (**C**) PVDF/0.5 wt% GO scaffolds. Reproduced with permission from [81], published by Elsevier, 2019.

Very recently, Abzan et al. adopted NIPS to fabricate three-dimensional piezoelectric PVDF and PVDF-GO scaffolds for nerve tissue engineering applications [81,106]. As recognized, GO possesses a range of oxygenated functional groups such as epoxy, hydroxyl, carbonyl and carboxyl. Carbonyl and carboxyl groups are mainly attached at the edge of basal plane of graphene sheet and hydroxyl groups on the plane. GO is produced by chemical oxidation of graphite flakes in strong oxidizing solution containing sulfuric acid, sodium nitrate, and potassium permanganate [114,115]. So hydrophilic oxygen functional groups of GO can render hydrophobic PVDF with improved hydrophilicity and enhanced GO-PVDF interfacial interactions. Accordingly, the incorporation of 0.5–3 wt% GO into PVDF reduces its water contact angle, especially with the 3 wt% GO addition. The water contact angle of hydrophobic PVDF is 117.2° ± 5.4°, but reduces markedly to 71.3° ± 6.4° by adding 3 wt% GO. As a result, PVDF/3 wt% GO scaffold is hydrophilic, thus favoring the attachment and growth of PC12 nerve cells. Moreover, GO additions also facilitate the formation of β-PVDF phase due to the interactions between carbonyl group (C=O) of GO and fluorine group (>CF_2_) of PVDF. The β-phase content in neat PVDF, PVDF/0.5 wt% GO (P-0.5GO), PVDF/1 wt% GO (P-1GO) and PVDF/3 wt% GO (P-3GO) scaffolds determined from Equation (1) is 64.5, 77.4, 64.6 and 70.15%, respectively. Apparently, GO additions can induce a high amount of β-phase in PVDF, leading to enhanced piezoelectric effect. The piezoelectric response can be determined by applying a mechanical force through a human finger imparting on the PVDF-based scaffold sandwiched between the copper electrodes (Figure 8a). The generation of average electrical voltage on neat PVDF, P-0.5GO, P-1GO, P-3GO and P-5GO scaffolds is 1.36 V, 1.78 V, 1.40 V, 1.49 V and 1.58 V, respectively (Figure 8b).

### 3.1. Electrospinning

**Figure 9:**
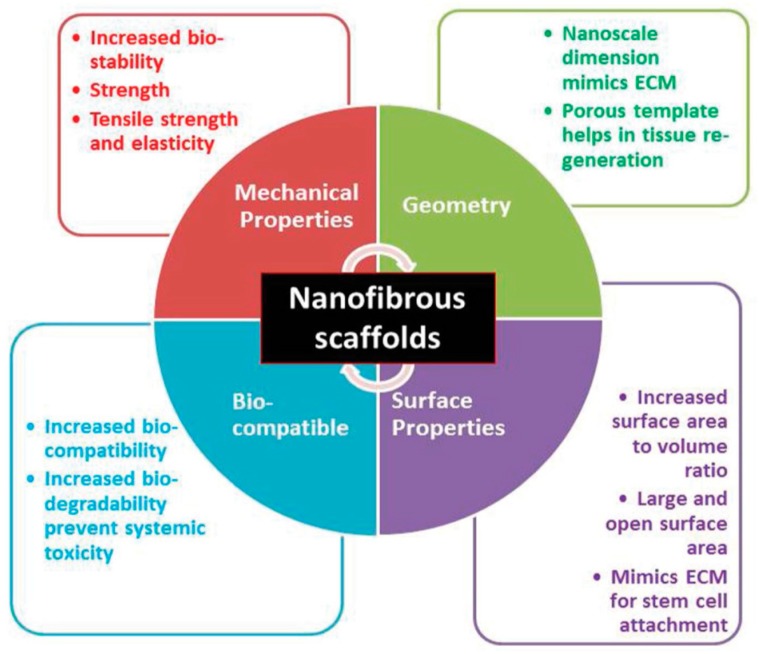
Schematic representation of the required properties of nanofibrous scaffolds including geometry, mechanical competence, biocompatibility and surface behavior.

**Figure 10:**
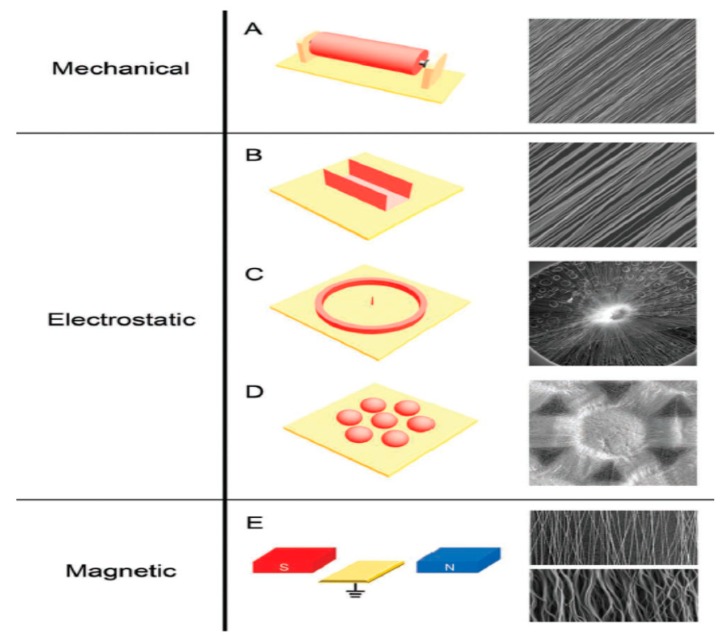
Schematics of electrospinning methods to direct fiber orientation by means of mechanical rotation of mandrel (**A**), electrostatic forces through the use of a metallic staple (**B**), a metallic ring (**C**), and an array of metallic beads (**D**), as well as magnetic forces through the use of a pair of permanent magnets (**E**). The yellow plates are grounded conductive electrodes. Scanning electron micrographs in the right panel show the morphologies of aligned nanofibers depositing at the target via different methods. Reproduced with permission from [120], published by Wiley-VCH, 2012. Reproduced with permission from [121], published by American Chemical Society, 2010.

Compared to conventional processed scaffolds, electrospun nanofibrous mats with a large surface to volume ratio are attractive for tissue engineering applications because they mimic the fibrillar structure of natural ECM secreted by the cells (Figure 9) [116]. Fibers from nano- to micrometer scale can be prepared by electrospinning. The fabrication process involves the application of a high electric field to the polymer/solvent solution. Above a critical voltage, electrostatic repulsion overcomes the surface tension of polymer droplet developed at a needle tip attached to the syringe pump. Therefore, a charged polymer jet is ejected from the needle tip towards a grounded collector, leading to the formation of random or aligned fibers. The electrically charged jet experiences bending instability and whipping motion resulting in a randomly oriented fiber deposition on the collector. Moreover, the jet also undergoes stretching due to electric field and solvent evaporation, thus causing jet thinning. The fiber diameter and porosity of the fibrous scaffolds depend on the processing parameters such as applied voltage, solution flow rate, type of solvent, polymer concentration in the solution, solution conductivity, and the distance between the needle and collector [117,118,119]. Because of the chaotic trajectory of the polymer jet, the fibers collected on a grounded collector generally exhibit random orientation, so depositing as a non-woven scaffold. Electrospun fibers can be aligned by controlling the rotation speed of the mandrel, magnetic field or electric field at the gap between a pair of electrodes (Figure 10) [118,120,121]. 

#### 3.1.1. Electrospun PVDF Scaffolds

**Figure 11:**
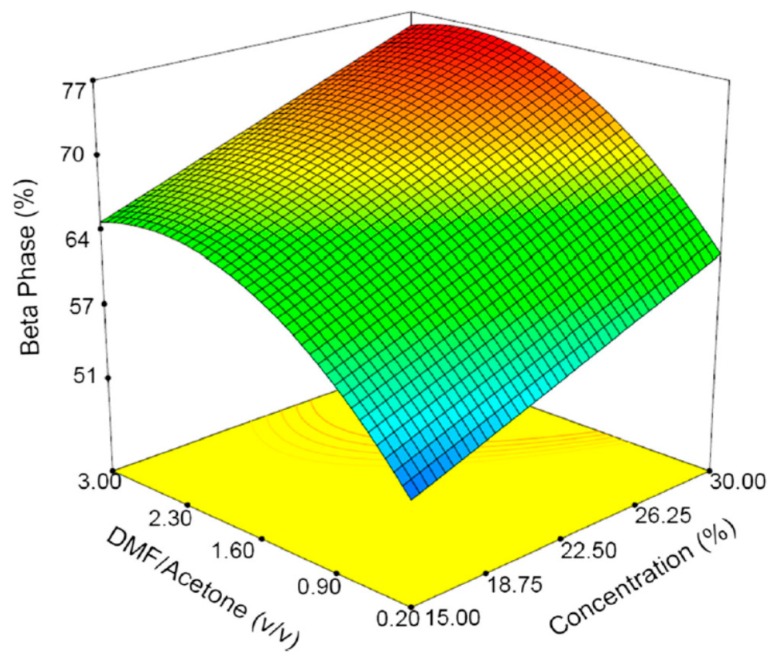
Three-dimensional plots of beta phase content vs. PVDF concentration and DMF/acetone ratio. Reproduced with permission from [124], published by Springer, 2018.

**Figure 12:**
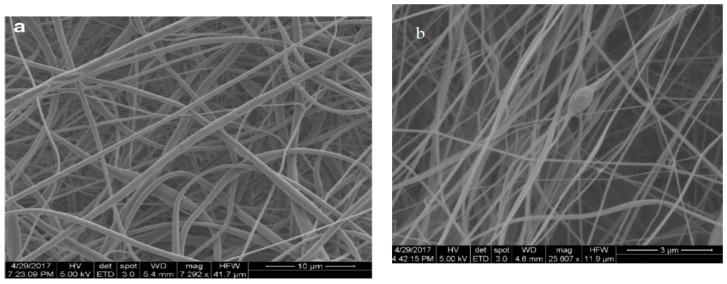
Scanning electron micrographs of electrospun nanofibers with a PVDF concentration of 25% and (**a**) DMF/acetone ratio of 1, and (**b**) DMF/acetone ratio of 3. Reproduced with permission from [124], published by Springer, 2018.

As mentioned above, piezoelectric properties of β-PVDF facilitate the generation of electrical potential on its surface due to mechanical deformation. The stretching of the polymer jet induces the β-PVDF phase, i.e., transforming nonpolar α-phase into polar β-phase [17,122,123,124,125]. So piezoelectric β-PVDF fibrous scaffolds that generate electrical stimulation are particularly attractive for bone and neural tissue engineering applications [15,17,46,125,126]. From the literature, existing studies are mainly focused on the effect of electrospinning parameters on the fiber morphology, fiber diameter and the β-phase formation. These parameters include PVDF concentration, solvent type, applied voltage, spinning distance, stationary or rotating collector, etc. [17,122,123,124,125,126,127,128]. In general, the fiber diameter increases with increasing PVDF concentration due to the higher solution viscosity and stronger intermolecular interactions. The selection of the appropriate solvent is crucial for forming smooth continuous fibers without beads. Volatile solvents with low boiling point and fast evaporation rate are generally preferred because they facilitate dehydration and solidification of the polymer jet. However, highly volatile solvents with very low boiling points can lead to the clogging and obstructing the flow-rate of polymer solution [128]. Apparently, smooth continuous PVDF fibers can be achieved by selecting a particular solvent combination, i.e., *N*,*N*-dimethylformamide (DMF; boiling point: 152 °C) and acetone (boiling point: 56 °C), or *N*,*N*-dimethylacetamide (DMAC; boiling point: 165 °C) and acetone. The slower evaporation rate of DMF or DMAC enables polymer jets to stretch and facilitate the transformation of α-phase to β-phase, whereas acetone can prevent the bead formation [17]. By adding more acetone (low DMF/acetone ratio), the evaporation rate of the polymer/solvent solution tends to increase, resulting in the formation of more α-phase in PVDF mats. In contrast, higher DMF/acetone ratios favor the formation of β-PVDF fibers with fined diameters. Figure 11 summarizes the 3D plots showing the β-phase content induced in electrospun fibrous mats as a function of PVDF solution concentration and DMF/acetone ratio. Typical SEM images of PVDF fibers prepared at different DMF/acetone ratios under a PVDF concentration of 25% are shown in Figure 12a,b.

**Figure 13:**
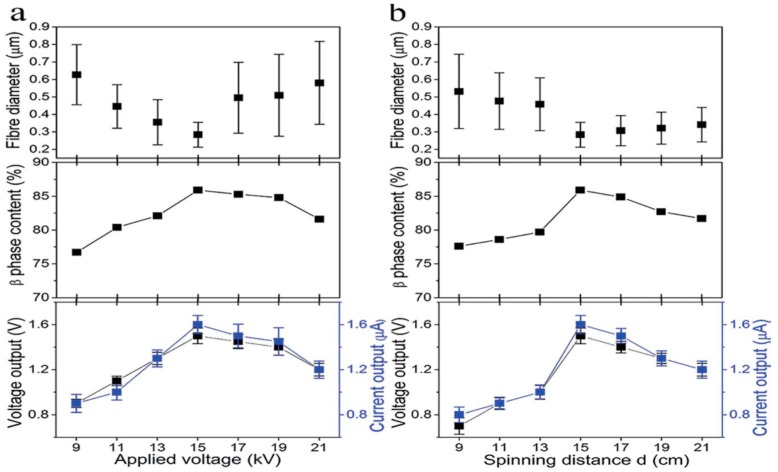
Effects of (**a**) applied voltage and (**b**) spinning distance on the fiber diameters, β-phase contents and electrical outputs of PVDF nanofiber mats (PVDF concentration 20%; nanofiber mat thickness 100 µm). Reproduced with permission from [129], published by Royal Society of Chemistry, 2015.

Recently, Shao et al. studied the processing-structural relationship of PVDF nanofibers prepared by electrospinning [129]. Increasing the applied voltage from 9 to 15 kV at a 20% PVDF solution leads to a higher charge density or electrostatic force for stretching the polymer jet, thereby decreasing the fiber diameter. Above 15 kV, the increase in fiber diameter is caused by the intensive bending instability (Figure 13a). The β-phase increases from 76.7% to 85.9% by increasing the applied voltage from 9 kV to 15 kV. Above 15 kV, the β-phase content decreases slowly with increasing applied voltage. Lanceros-Méndez and coworkers also demonstrated that the applied voltage can produce a higher stretching of the polymer jet, favoring the formation of β-phase [123]. In addition, the strong electric field employed also plays the role of fiber poling. A strong dependence of the fraction of β-phase in PVDF fibers on the applied voltage has also been reported by Sengupta et al. [130]. From Figure 13a, electrical (voltage/current) outputs of electrospun mats follow the increasing/decreasing trend of the β-phase content with the applied voltage. The fiber size and β-phase content of PVDF fibrous scaffolds also depend on the spinning distance (needle tip to collector distance) (Figure 13b). A long spinning distance offers a large space for jet stretching and more time for solvent evaporation, thus giving rise to fibers with fined diameters. For a 20% PVDF solution under an applied voltage of 15 kV, the fiber diameter decreases from about 550 nm to 458 nm as the spinning distance increases from 9 cm to 13 cm. So coarse fibers are produced at such short distances. At a spinning distance of 15 cm, the fiber size decreases significantly to 284 nm. This is a critical distance range to form nanofibers with fine diameters. Accordingly, the highest β-phase content of 85.9% is achieved at this critical distance. The voltage/current outputs of PVDF fibrous follow a similar changing trend of the β-phase content with the spinning distance.

##### Uniaxially Aligned Nanofibers

Electrospun nanofibers can be aligned into uniaxial arrays through mechanical rotation of the collector (e.g., drum, disk), the gap method and magnetic electrospinning [118,120,121,131]. From the literature, several research groups have prepared aligned PVDF fibers by rotating a collecting drum at various speeds due to its simplicity [15,132,133,134]. For instance, Lins et al. classified the alignment of PVDF fibers obtained at different drum speeds as non-aligned (50 rpm), low-aligned (1000 rpm), medium-aligned (2000 rpm) and high-aligned (3000 rpm) on the basis of SEM observations [15]. Apparently, a drum speed of 50 rpm is relatively low to initiate the fiber alignment. The fiber orientation of electrospun scaffolds increases with increasing drum speed from 1000 to 3000 rpm. Increasing rotating speed also reduces the fiber diameter and porosity of the scaffolds. The average diameter of PVDF fibers under rotating speeds of 50, 1000, 2000, and 3000 rpm is 1.98 ± 0.55, 1.95 ± 0.60, 1.56 ± 0.60, and 1.51 ± 0.68 µm, respectively. The porosity level of scaffolds fabricated under these speeds is 86, 79, 54 and 46%, respectively. Thus, fiber alignment results in a decrease in the porosity, but an increase in the pore size. The pore size of highly aligned (300 rpm) scaffold is 8.5 ± 3.2 µm, while that of non-aligned (50 rpm) scaffold is 4.2 ± 0.4 µm. The aligned PVDF nanofibers also contain the β-phase as expected. Wu et al. demonstrated that the β-phase content of aligned PVDF fibers reaches 88% by rotating a drum collector, being much higher than that of randomly oriented PDVF fibers deposited on a stationary collector (79%) [134]. 

**Figure 14:**
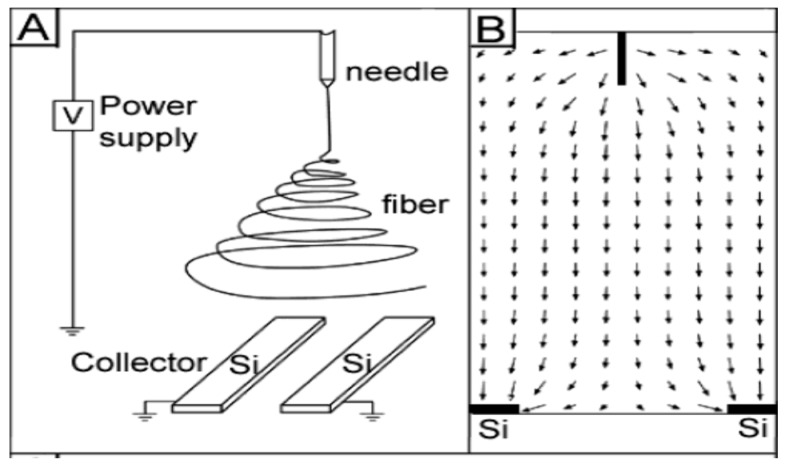
Schematic diagram showing electrospinning setup for forming uniaxially aligned nanofibers. (**A**) A collector with two pieces of conductive silicon stripes separated by a gap. (**B**) Electric field strength around the needle and the collector. The arrows denote the direction of the electrostatic field lines. Reproduced with permission from [135], published by American Chemical Society, 2003.

**Figure 15:**
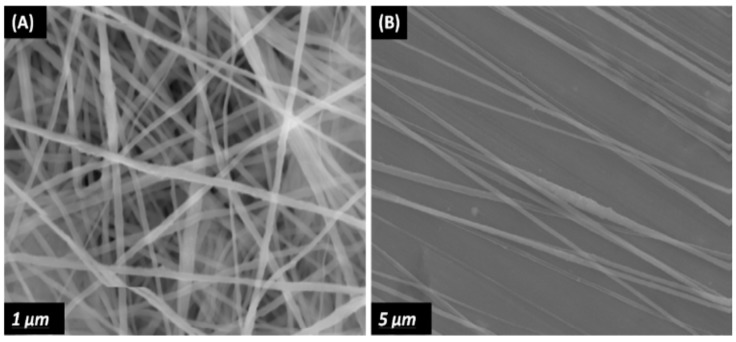
(**A**) Randomly oriented PVDF nanofibers deposited on a stationary metal plate covered with aluminum foil. (**B**) Aligned PVDF nanofibers deposited at the gap between two metallic bars of a collector.

In general, the rotating collector method is a time-consuming process, and requires proper manipulation of the speed to ensure the as-spun nanofibers with desired properties. In contrast, the gap method is an effective route to fabricate uniaxially aligned arrays by using two parallel conductive silicon strips or bars separated by a gap acting as a collector. In this context, the fibers can be aligned in the gap between two conducting electrodes (Figure 14) [135]. In the process, the electric field lines in the vicinity of the collector are split into two directions toward the opposite edges of the gap. Thus, the charged nanofibers are pulled toward both edges of the conductors, and stretched across the gap to form a parallel, uniaxial array mat. Very recently, Shebata et al. prepared aligned PVDF and PVDF/MWCNT fibrous mats using the gap method. They reported that the two-bar collector system provides a superior alignment for the PVDF nanofibers [136]. Figure 15a,b shows typical SEM images of randomly oriented and aligned PVDF nanofibers. Randomly oriented fibers are deposited on a grounded metal plate covered with aluminum foil.

##### PVDF Nanocomposite Scaffolds

The incorporation of functional nanoparticles into PVDF has a large influence on its structural properties, especially conducting nanomaterials such as MWCNTs, graphene sheets and silver nanoparticles (AgNPs). It is well established that the conductivity of polymer solutions increases dramatically by adding an appropriate salt such as sodium chloride. The salt increases the number of ions in the polymer solution, thereby enhancing surface charge density of the polymer solution and the electrostatic force generated by the applied electric field [127,128,137]. This leads to spun fibers with fine diameters. As recognized, nanoparticle additions to the polymer solutions can increase their viscosity and fiber diameter. For conducting AgNPs and MWCNTs, there exists a balance between the increase in solution conductivity and solution viscosity. In recent years, graphene derivatives such as graphene oxides and reduced graphene oxide (rGO) have found potential biomedical applications for making biosensors, tissue engineering scaffolds and orthopedic implants [138,139]. Graphene oxide is an electrical insulator due to the presence of oxygen functional groups. Its electrical conductivity can be resumed by either treating with reducing agents such as hydrazine and sodium borohydride to form reduced graphene oxide (rGO) [140], or via rapid heating in a furnace at 1050 °C to generate thermally reduced graphene oxide (TRG) [141]. Existing literature reports reveal that AgNPs, rGO or MWCNT nanomaterials are very effective for inducing the β-phase in electrospun PVDF fibrous mats by serving as the nucleating sites [92,142,143]. Therefore, both the conducting nanofillers and electrostatic field stretching during electrospinning contribute to the formation of β-PVDF. 

As is widely recognized, piezoelectricity is reversible, because mechanical energy can be converted to electrical energy and vice versa. The strain S_j_ induced in a piezoelectric material by an applied electric field E can be written as [144],

S_j_ = *d*_ij_·E_i_ (2)

where *d*_ij_ is the piezoelectric coefficient; the subscript indices 1–3 describe components along the x, y, and z axis of a rectangular coordinate system, and indices 4–6 represent shear components of the strain. Accordingly, the longitudinal piezoelectric coefficient (*d*_33_) can be determined from the following Equation:

S = *d*_33_·E (3)

So *d*_33_ describes the strain induced by an applied electric field (E_3_; V/m), having a unit of pm/V. An alternate way to express *d*_33_ is as an induced polarization in the direction 3 (C/m^2^) per unit applied stress (N/m^2^), yielding an equivalent unit of pC/N [145]. PVDF and P(VDF-TrFE) generally show an unusual negative longitudinal piezoelectric effect, i.e., a contraction in their lattice constants under the application of an electric field. Therefore, a negative sign is often used to describe such a parameter [146].

Bose and coworkers fabricated electrospun PVDF, PVDF/1 wt% MWCNT and PVDF/1 wt% (MWCNT-AgNP) fibrous mats with randomly oriented fibers [147]. Carboxyl functionalized MWCNT and AgNP decorated MWCNT were added to PVDF for enhancing its piezoelectric effects. The incorporation of both types of MWCNTs into electrospun PVDF fibers led to an increase in the β-phase content. As a result, the *d*_33_ value of the PVDF/1 wt% MWCNT and PVDF/1 wt% (MWCNT-AgNP) fibrous mats increased markedly, especially the latter, with MWCNT-AgNP nanofillers (Table 1). In a recent study, Bose and coworkers employed carboxylated GO (CGO) and fluorinated GO (FGO) nanofillers for further enhancing *d*_33_ value of PVDF fibrous mats. The highest β-phase content was achieved in the PVDF/1 wt% FGO mat due to the presence of highly electronegative fluorine [148]. Consequently, PVDF/1 wt% FGO fibrous mat had a high *d*_33_ value of 63 pm/V. So the addition of very low loading level of GO-based nanofillers into electrospun PVDF scaffolds led to enhanced piezoelectricity. Combining GO addition with electrospinning offers the opportunity to fabricate PVDF fibrous mats with tunable piezoelectric characteristics. Very recently, Wu et al. deposited aligned PVDF and PVDF/MWCNT fibers on a rotating drum, and randomly oriented PDVF fibers on a stationary collector, respectively. They then determined *d*_33_ values of electrospun PVDF fibers with different orientations under mechanical force deformations [134]. Compared to random fibers, aligned PVDF/MWCNT fibers exhibited the largest *d*_33_ value of 31.3 ± 2.1 pC/N due to the presence of the highest amount of the β-phase content (Table 1). 

#### 3.1.2. Electrospun P(VDF-TrFE) Scaffolds

As mentioned, the presence of extra fluorine atoms in the TrFE monomer means that the β-phase can crystallize easily from P(VDF-TrFE) melt without mechanical stretching. Additional annealing, mechanical stretching or electrical poling can lead to a further enhancement of the β-phase content. Generally, annealing is particularly useful for enhancing the degree of crystallinity, reducing the porosity and eliminating the residual solvent of the P(VDF-TrFE) films [149]. From an economic point of view, P(VDF-TrFE) is more expensive than PVDF, owing to the production risk of explosion of TrFE monomer during co-polymerization [150]. Nevertheless, P(VDF-TrFE) has found wide applications for energy harvesters [151,152], and bone tissue engineering [8].

**Figure 16:**
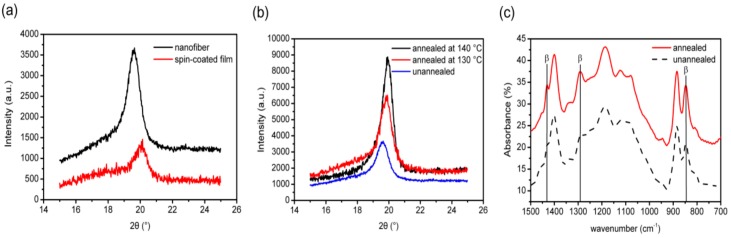
(**a**) X-ray diffraction (XRD) patterns of spin-coated P(VDF-TrFE) film and electrospun P(VDF-TrFE) nanofibers. (**b**) XRD patterns of the P(VDF-TrFE) nanofibers before and after annealing at 130 °C and 140 °C for 2 h. (**c**) FTIR spectra of the P(VDF-TrFE) nanofibers before and after annealing at 140 °C for 2 h.

Jiang et al. prepared aligned P(VDF-TrEF) fibers (400–550 nm) with a rotating drum under a rotation speed of 2500 rpm followed by annealing at 130 °C and 140 °C [153]. Furthermore, spin-coated P(VDF-TrEF) film was also fabricated for the purpose of comparison. The XRD pattern of the as-spun fibers revealed the presence of a strong characteristic peak at about 20°, corresponding to the (110) and (200) planes of the β-phase. This characteristic peak became more intense in the spun fibers by annealing at 130 °C and 140 °C (Figure 16a,b). FTIR spectra also showed that the intensity of the bands due to the β-phase (846, 1285 and 1431 cm^−1^) of the annealed sample was stronger than that of the same bands of the unannealed specimen, especially at 846 and 1285 cm^−1^ (Figure 16c). Therefore, the β-phase content was further increased by the annealing treatment. 

**Figure 17:**
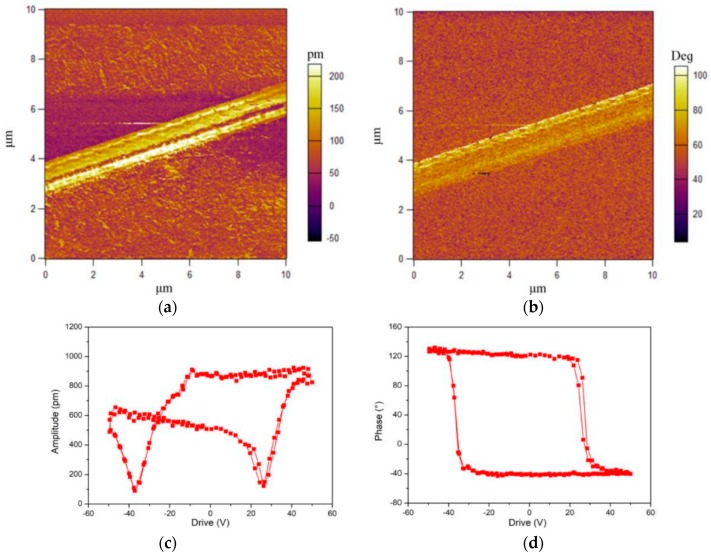
Piezoresponse force microscopy: (**a**) amplitude and (**b**) phase images of a single P(VDF-TrFE) nanofiber. (**c**) PFM amplitude and (**d**) PFM phase of the P(VDF-TrFE) nanofiber as functions of DC bias for two cycles, displaying good repeatability for forward and reverse scans.

Jiang et al. also employed piezoresponse force microscopy (PFM) to examine piezoelectric response of a single P(VDF-TrEF) fiber [153]. PFM is a contact-mode atomic force microscopy (AFM) in which the cantilever tip is used to detect piezoelectric response of a ferroelectric material. By applying an AC voltage to the cantilever tip, the material experiences expansion or contraction. So the piezoresponse of a sample can be determined through the deflection of the cantilever associated with the strain of mechanical deformation. The piezoresponse is detected and quantified in terms of its amplitude and phase by a lock-in amplifier. The PFM amplitude and phase signals characterize the magnitude of the piezoelectricity and the polarization direction, respectively [154]. Figure 17a,b shows the respective PFM amplitude and phase images of a single P(VDF-TrFE) nanofiber. The amplitude image reveals a strong piezoelectric contrast because of the deflection caused by the applied AC field. By superimposing a DC bias voltages of ±50 V, a butterfly-like amplitude loop and a well-defined hysteresis phase loop are generated, as shown in Figure 17c,d. The butterfly loop is a typical characteristic of piezoelectric materials [154]. So P(VDF-TrFE) nanofiber exhibits excellent piezoelectricity due to the presence of electroactive β-PVDF phase. 

**Figure 18:**
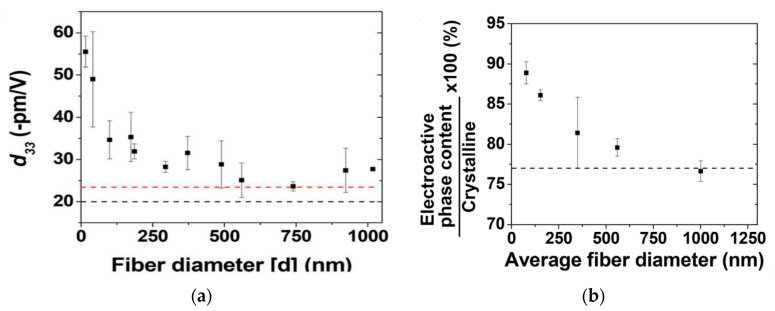
(**a**) Measured *d*_33_ as a function of fiber diameter from PFM. The red dashed line corresponds to the measured *d*_33_ of a 80 µm thick film, and the black dashed line is the *d*_33_ of bulk P(VDF-TrFE). Thick P(VDF-TrFE) film (80 µm) was made by drop-casting and employed as a reference. (**b**) Electroactive phase content determined by FTIR as a function of mean fiber diameter. The black dashed line represents the measured electroactive active content of a thick film prepared by drop-casting. Reproduced with permission from [152], published by Royal Society of Chemistry, 2016.

Ico et al. prepared electrospun P(VDF-TrFE) mats with fiber diameters ranging from 1 µm to nanometer scale [152]. Such a fiber size reduction led to an increase of electroactive phase content and the degree of crystallinity. Furthermore, they also employed PFM to determine the *d*_33_ value of a single P(VDF-TrFE) fiber. The *d*_33_ value of electrospun P(VDF-TrFE) fiber was found to be size dependent, and reached 55 pm/V for the fiber with a few nanometer size. Thus, a reduction of fiber diameters of P(VDF-TrFE) from micro- to nanoscale dimensions resulted in higher piezoelectricity (Figure 18a). The fiber size dependency of piezoelectric coefficient correlated well with the amount electroactive β-PVDF phase induced in the fibrous mat during electrospinning. The amount of β-phase induced was also dependent on the fiber diameter. The β-phase content determined from the FTIR spectroscopy increased considerably as the fiber diameter reduced to <100 nm (Figure 18b).

#### 3.1.3. Melt Electrospinning

Electrospun fibrous mats can be prepared either using polymer solution or melt. Solution electrospinning has a main drawback involving the use of organic solvents to dissolve polymers. Most organic solvents are harmful to biological cells and may cause detrimental effects in tissue engineering applications. In this respect, electrospinning of polymer melts offers an advantage for fabricating fibrous structures without using organic solvents. Melt-electrospinning has been studied to a significantly lesser extent than solution electrospinning, because this process results in larger fiber diameters of several to hundreds of micrometers [155]. In the process, polymer melts with high viscosity and low conductivity suppress whipping motion, allowing the jet travels in a straight path from the needle to the collector. So the melt viscosity plays a crucial role in determining the spinnability and fiber diameter of a polymer. In general, low melt viscosity would lead to the formation of beads. At a very high melt viscosity, the electric field cannot overcome the viscosity resistance for spinning [156]. The melt viscosity of polymers is controlled by their molecular weight and heating temperature. The electrical heating system is the most commonly used for melting polymers. However, the electrical heating source can interfere with the high-voltage spinning system. Accordingly, laser heating has some advantages, such as fast melting of polymers due to intense laser beams, and noninterference with the high-voltage spinning system. The melt-electrospun studies in the literature are focused mainly on the fabrication of polycaprolactone (PCL) fibers [157,158,159].

**Figure 19:**
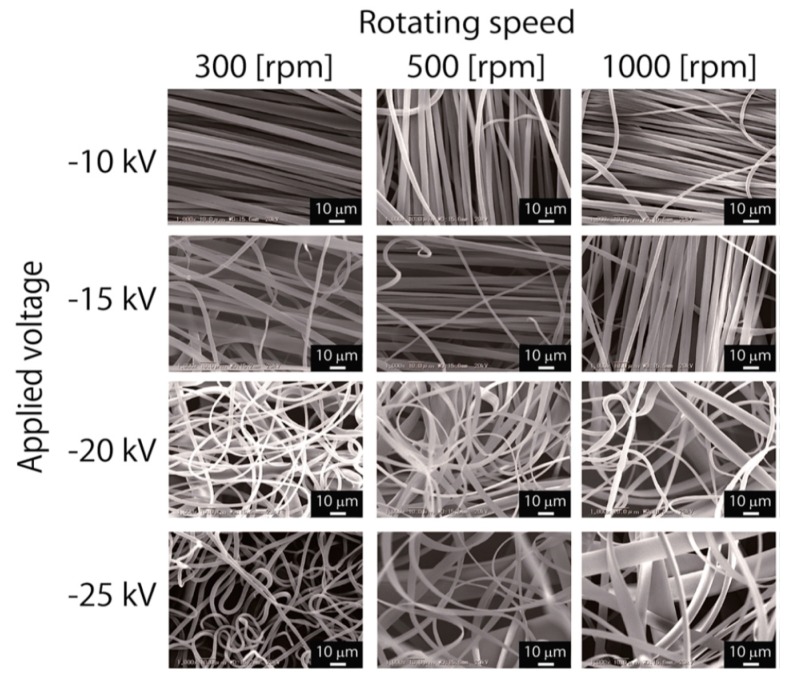
SEM images of PVDF fibrous mats prepared by melt-electrospinning at various applied voltages and collector rotating speeds. The laser output power and the polymer feed rate were fixed at 53 W and 1 mm min^−1^, respectively. Reproduced with permission from [160], published by Royal Society of Chemistry, 2017.

Very recently, Asai et al. prepared electrospun PVDF fibers by means of laser-melt electrospinning (MES) [160]. In the MES approach, PVDF was melted by a CO_2_ laser beam, and the molten jet was accelerated at different applied voltages towards a rotating collector of various speeds. Figure 19 shows the SEM images of PVDF fibers formed at different applied voltages and collector rotation speeds. Aligned PVDF fibers formed only at a low voltage of −10 kV for different rotating speeds. The average diameter of fibers spun at −10 kV and 1000 rpm was 4.2 ± 3.4 µm. By increasing the voltage to −25 kV and collector speeds to 500–1000 rpm, ribbon-like flat fibers were produced. The XRD results revealed that non-polar α-phase was a dominant structure in the PVDF fibers. As such, melt-electrospun PVDF fibers had a very low *d*_33_ value of 0.03 pC/N.

## 4. In Vitro and In Vivo Models

### 4.1. Bone Tissue Engineering

Human mesenchymal stem cells (hMSCs) can differentiate into osteoblasts, adipocytes and chondrocytes. Osteoblasts produce bone matrix proteins and mineralize the matrix into bones. Preosteoblast differentiation proceeds through 3 stages including proliferation, extracellular matrix maturation, and mineralization [161]. In the first stage, osteoblasts proliferate by secreting bone matrix proteins, such as collagen type 1 alpha 1 (Col1α1), osteopontin and fibronectin. In stage 2, they begin to differentiate and mature the ECM with alkaline phosphatase (ALP) and collagen. The final bone matrix mineralization occurs by expressing osteocalcin, leading to the deposition of calcium phosphate [162]. 

Human bones have the ability of self-remodeling through an electromechanical mechanism due to the piezoelectric effect [163]. Thus, mechanical stimulation of bones induces their growth and regeneration as a result of the generation of electrical potential [3,23]. The application of electrical stimulation has been found to be effective in enhancing rat bone marrow mesenchymal stem cells (rBMSCs) and rat adipose-derived mesenchymal stem cells (AT-MSCs) migration, proliferation, differentiation, and stimulates high levels of osteogenic expressions. Electrically stimulated rBMSCs and AT-MSCs exhibit an increased osteogenic differentiation, as indicated by high expression levels of osteogenic markers, including collagen 1, osteopontin, Runx 2 and calmodulin [164,165]. Furthermore, electrical stimulation of AT-MSCs also promotes bone repair and bone regeneration in vivo as manifested by implanted scaffolds with AT-MSCs in the rat femur defects [166]. Thus, human MSCs capable of differentiating into osteoblasts show great potential for healing of bone defects [167].

**Figure 20:**
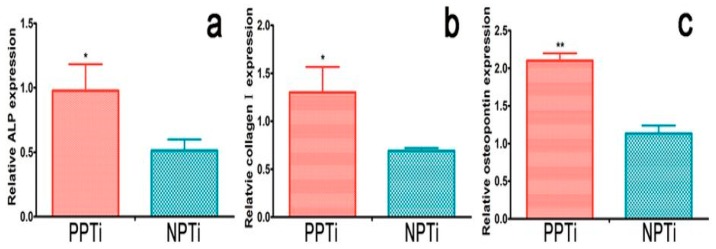
(**a**) Relative ALP, (**b**) collagen I, and (**c**) osteopontin gene expressions of rBMSCs cultured on PPTi and NPTi samples for 14 days (*n* = 3). * *p* < 0.05, ** *p* < 0.01 compared with NPTi.

Electroactive PVDF can be used for modifying the surface of titanium in order to improve its bioactivity. Titanium (Ti) and its alloys are widely used as load-bearing implant materials in orthopedics. Polarization of the PVDF film coated on Ti has been reported to promote osteoblastic cell adhesion and proliferation [2]. More recently, Zhou et al. deposited PVDF film on Ti through corona discharge at 100 °C. They demonstrated that polarized PVDF-Ti sample promotes osteogenic differentiation of rBMSCs [161]. Figure 20 shows the real-time polymerase chain reaction (RT-PCR) analysis, revealing the expression of osteogenic differentiation-related genes of rBMSCs on polarized PVDF-Ti (PPTi) and nonpolarized PVDF-Ti (NPTi) samples. Apparently, the cells on the PPTi sample exhibit significantly higher gene expression than those on the NPTi after 14 days of incubation. This is due to the presence of surface charges on PPTi as a result of the polarization.

#### 4.1.1. In Vitro Cell Cultivation

**Figure 21:**
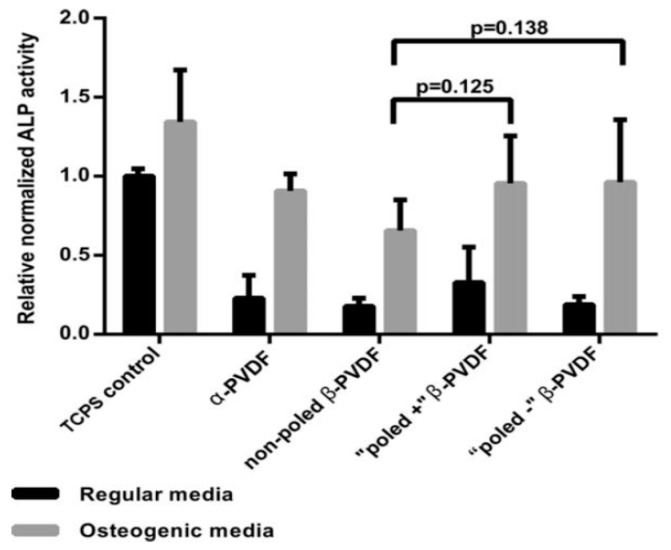
Relative alkaline phosphatase activity of hASCs on different PVDF films and tissue culture polystyrene (TCPS) control. The ALP activity was normalized against the DNA content of the cells. Reproduced with permission from [171], published by Wiley, 2015.

Lanceros-Méndez and coworkers carried out a series of studies on the interactions between the PVDF and cells [13,48,105,112,168,169]. In those studies, solvent-cast dense films, solvent-cast particulate-leaching films, and NIPS porous membranes were fabricated. Particular attention was paid to the effect of the surface charge of PVDF induced by electrical poling on the fibronectin adsorption, osteoblastic cell attachment and proliferation [13,168]. Solvent-cast PVDF films (α-PVDF) were mechanically drawn to induce β-PVDF. The β-PVDF films were further poled by corona discharge to induce negative and positive electrical surface charge on the cell culture side, respectively [168]. The results showed that electrical poling decreased the water contact angle of β-PVDF films. The dipoles of β-PVDF would interact with water molecules, thereby enhancing their wettability. The positively and negatively poled β-PVDF films exhibited a water contact angle of 31.8° and 51.1°, respectively, thus showing hydrophilic behavior. Therefore, poled β-PVDF films favored fibronectin protein adsorption, thereby facilitating MC3T3-E1 osteoblastic cell adhesion and proliferation. It is generally known that the surface hydrophilicity and topography modulate protein adhesion, thereby affecting cellular response accordingly [170]. Apparently, the combination of surface wettability and piezoelectricity was effective for promoting osteoblastic cell attachment and proliferation. In another study, it was also found that the differentiation of human adipose-derived stem cells (hASCs) into osteogenic lineage was affected by substrate polarization of β-PVDF. Negatively poled β-PVDF promoted higher osteogenic differentiation, as evidenced by higher ALP activity (Figure 21) [13,171]. 

##### Electrospun Fibrous Scaffolds 

Arinzeh and coworkers studied the beneficial effect of piezoelectric scaffolds for tissue engineering applications [172,173]. They prepared nonwoven PVDF fibrous mats by using electrospinning under applied voltages of 12–30 kV [17]. The fibrous mat formed at 25 kV had the highest 72% β-phase, while the mat fabricated at 12 kV contained 68% β-phase. Therefore, hMSCs cultivated on the PVDF-25 kV scaffold had higher levels of ALP activity and biomineralization when compared to the PVDF-12 kV mat. They also studied osteogenic differentiation of hMSCs on nonwoven P(VDF-TrFE) fibrous mats under dynamic compression at 1 Hz with 10% deformation to mimic physiological strain conditions [14]. Two power sources were employed to fabricate P(VDF-TrFE) fibrous mats with a large thickness of 3 mm, porosity of 90% and 64% β-phase. These fibrous mats were further annealed at 135 °C to increase the degree of crystallinity and β-phase content (i.e., 75%). Osteogenic markers of ALP activity, mineralization, and osteocalcin of all mats were examined accordingly. They reported that the ALP activity and matrix mineralization of the as-spun P(VDF-TrFE) mat were considerably lower than those of annealed P(VDF-TrFE) and PCL control at day 28. More recently, Kitsara et al. treated electrospun PVDF nanofibrous scaffolds with oxygen plasma for improving their hydrophilicity [174]. As a result, osteoblasts cultivated on hydrophilic PVDF fibrous scaffolds had better cell spreading over the non-treated counterparts as expected. 

**Figure 22:**
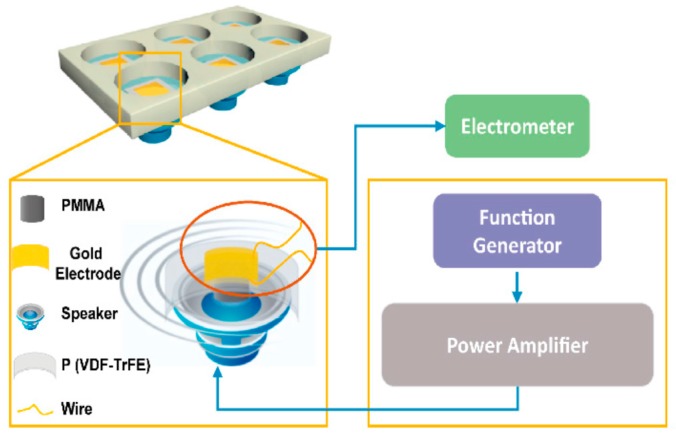
A simple set-up of flexible-bottomed culture plate together with an attached speaker for inducing mechanical vibration.

**Figure 23:**
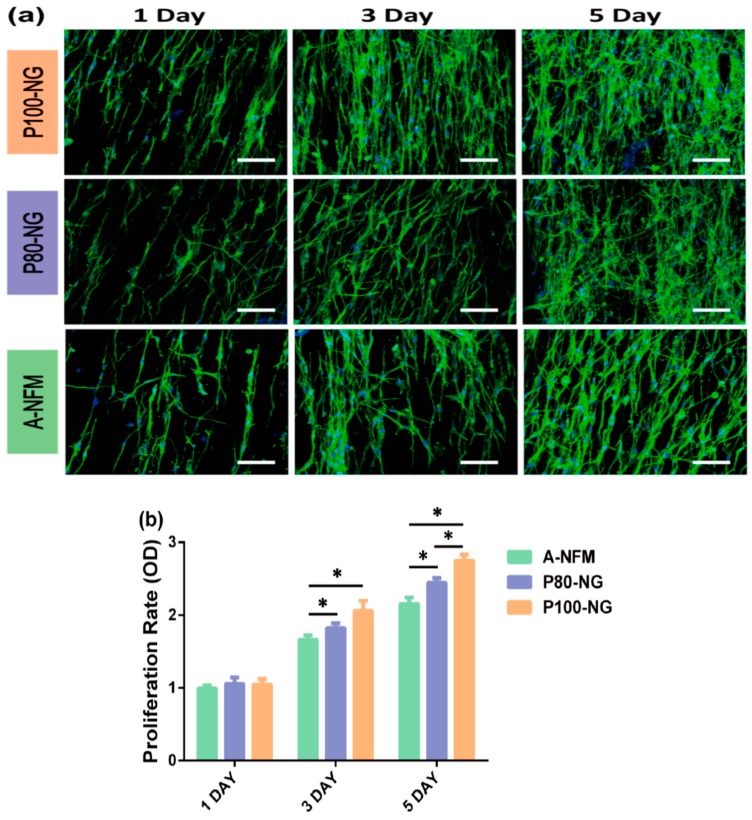
(**a**) Fluorescence micrographs of MC3T3 cells cultured on fibrous A-NFM, P80-NG, and P100-NG mats for 1, 3, and 5 days. The scale bar is 100 µm. (**b**) Proliferation of MC3T3 cells on P80-NG, P100-NG, and A-NFM mats. All data represent the mean standard deviation (n = 3, * *p* < 0.05).

Wang et al. fabricated electrospun P(VDF-TrFE) mats with aligned fibers followed by annealing and electrical poling. The mean diameter of electrospun nanofibers was 590 nm ± 26 nm [175]. The β-phase content of the as-spun, annealed and poled P(VDF-TrFE) mats was 43.1%, 46.6% and 69.2%, respectively. They studied the effect of dynamic electrical stimulation on the adhesion and proliferation of mouse osteoblastic cells (MC3T3-E1) on annealed P(VDF-TrFE) and electrically poled P(VDF-TrFE) mats. Annealed P(VDF-TrFE) was designated as A-NFM, and P(VDF-TrFE) poled with the electric field of 80 MV/m and 100 MV/m were labeled as P80-NG and P100-NG, respectively. The set-up of flexible-bottomed culture plate was given in Figure 22. A speaker attached to the bottom of the cell culture plate would generate mechanical vibration at a frequency of 2 Hz for mimicking low-frequency biomechanics. The CCK-8 assay was used to examine the proliferation of MC3T3-E1 osteoblasts. Fluorescence microscopy images of MC3T3 osteoblasts on A-NFM, P80-and P100-NG revealed that the cells were elongated and oriented along the direction of nanofibers (Figure 23a). Moreover, P100-NG and P80-NG exhibited a higher cell proliferation rate than A-NFM. So poled fibrous mats with piezoelectricity increased osteoblastic cell proliferation considerably (Figure 23b).

**Figure 24:**
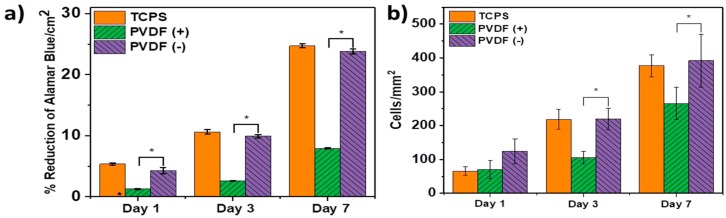
(**a**) Cell proliferation based on Alamar Blue assay of MG63 osteoblasts cultured on PVDF(+), and PVDF(−) scaffolds, as well as tissue culture polystyrene (TCPC) control for 1, 3 and 7 days. (**b**) Number of cells/mm^2^ growing on TCPS control, PVDF(+) and PVDF(−) fibers after cultivation of MG63 cells for 1, 3 and 7 days. * Significant difference between PVDF(+) and PVDF(−) samples determined with Tukey test (*p* < 0.05). Reproduced with permission from [10], published by American Chemical Society, 2019.

**Figure 25:**
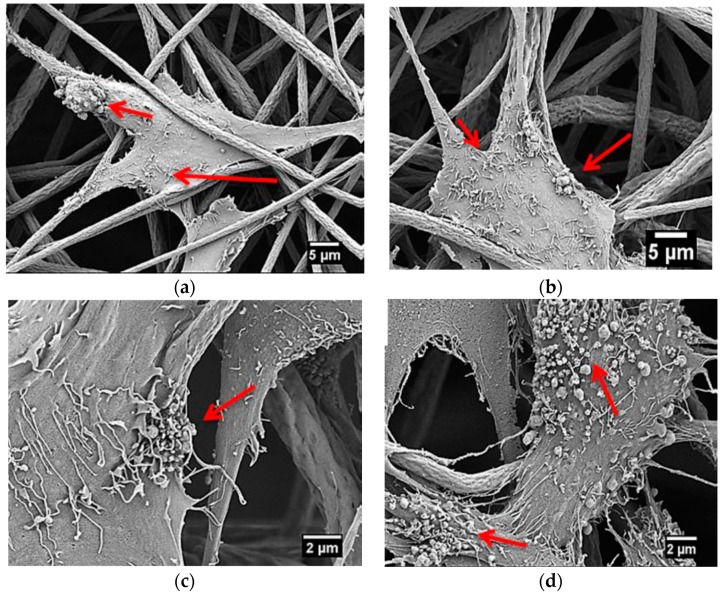
SEM images showing the formation of collagen fibrils on MG63 osteoblasts cultured on electrospun (**a**) PVDF(+) and (**b**) PVDF(−) scaffolds for three days. Accumulation of collagen fibrils and calcium phosphate nodules on MG63 osteoblasts cultured on (**c**) PVDF(+) and (**d**) PVDF(−) scaffolds for seven days. Red arrows indicate collagen fibrils present on cell surfaces. Reproduced with permission from [10], Copyright American Chemical Society, 2019.

Szewczyk et al. prepared PVDF(+) and PVDF(−) fibrous scaffolds by applying a constant voltage of 15 kV with positive and negative polarities to the stainless needle during electrospinning. Human osteoblast-like cell line (MG63) was then cultivated on those scaffolds [10]. The surface potential value of the PVDF(+) and PVDF(−) fibers was −173 mV and −65 mV, respectively as determined by the Kelvin probe force microscopy. Increased cell viability/proliferation was found in the PVDF(−) samples at different time points on the basis of Alamar Blue results (Figure 24a). Furthermore, PVDF(−) fibers also exhibited a much higher number of cells in comparison with the PVDF(+) fibers (Figure 24b). They attributed this to the surface potential of PVDF(−) fibers (i.e., −65 mV) was very close to the membrane potential of MG63 cell having a value of −60 mV. Collagen fibrils with an average diameter of 0.15 μm were readily seen on the osteoblasts cultured on the PVDF(+) and PVDF(−) fibers (Figure 25a,b) for three days. At day 7, small round nodules were observed on the surfaces of osteoblasts cultured on both scaffolds. However, the PVDF(−) fibers had a higher density of round nodules (Figure 25c,d). Those nodules were calcium phosphate as evidenced by the presence of Ca and P signals in the X-ray energy dispersive spectrum, thus showing collagen mineralization. Accordingly, the formation of mineralized collagen fibrils on the PVDF fibers can be tailored by monitoring the surface potential of electrospun scaffolds.

It is noteworthy that the incorporation of nanoparticles (e.g., ZnO, GO and barium titanate) into electrospun PVDF-based scaffolds enhances the adhesion, proliferation and differentiation of hMSCs [176,177,178]. This is because those nanoparticles serve as effective nucleating sites for forming electroactive β-phase [77]. Augustine et al. reported that ZnO nanoparticles have a positive influence on the cellular behavior of electrospun P(VDF-TrFE) scaffolds. Both the hMSCs and human umbilical vein endothelial cells (HUVECs) cultivated on P(VDF-TrFE)/ZnO nanocomposite scaffolds showed higher cell adhesion and proliferation compared to the same cells cultured on pure P(VDF-TrFE) scaffolds [177]. Saburi et al. demonstrated that GO nanofillers of electrospun PVDF-GO nanofibers enhance osteogenic differentiation of human induced pluripotent stem cells (iPSCs). The ALP, Runx2, osteocalcin and osteonectin gene expression levels of the iPSCs cultivated on the PVDF-GO fibrous scaffold were significantly higher than those cultured on neat PVDF fibrous mat [178].

#### 4.1.2. In Vivo Models for Bone Tissue Engineering

**Figure 26:**
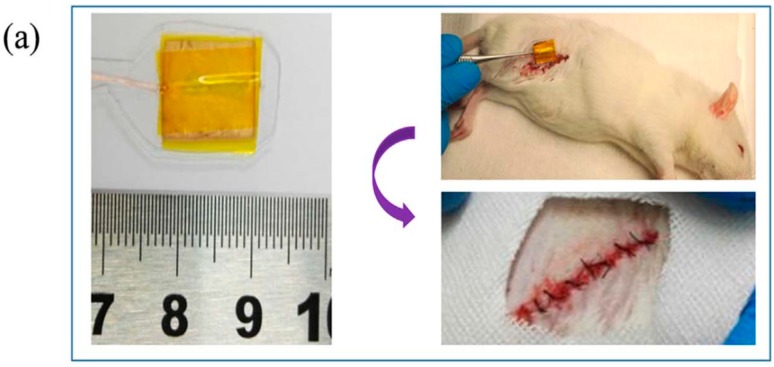

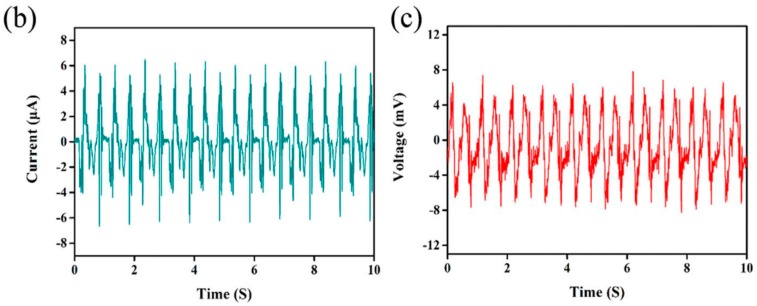
(**a**) Photographs showing the dimension of a poled P(VDF-TrFE) scaffold before implantation (left), the process of implanting piezoelectric scaffold into subcutaneous thigh region of a SD rat (upper right), and the implanting site after suturing (lower right). (**b**) Current and (**c**) voltage outputs of electrospun P(VDF-TrFE) nanofibrous scaffold after implantation under pulling. Reproduced with permission from [180], published by Elsevier, 2018.

In vivo animal models can be used to assess biocompatibility of polymeric materials that directly contact with different living tissue types including nerve, bone and blood. Compared to in vitro cell culture studies, limited information is available on the animal bone tissue responses to piezoelectric PVDF-based scaffolds in vivo [39,177,179,180]. Guo et al. performed subcutaneous implantation of PVDF/polyurethane (1:1, *v*/*v*) scaffolds into Sprague-Dawley (SD) rats. They reported that such scaffolds showed higher fibrosis level due to the piezoelectric stimulation as a result of random rat movements followed by mechanical deformation of the scaffolds [39]. Lanceros-Méndez and coworkers implanted non-poled β-PVDF films, poled β-PVDF films (*d*_33_ = −24 pC/N) and randomly oriented β-PVDF fibrous mats into bone defects created in each femur of Wistar rats [179]. After 4 weeks, the femurs were removed from the sacrificed rats and subjected to histological examinations. Bone defects treated with randomly oriented β-PVDF fibers displayed obvious bone regeneration. This was revealed by the presence of organized fibers and trabecular formation. Moreover, poled β-PVDF films also showed the formation of bone marrow and trabecular bone. Very recently, Wang et al. investigated piezoelectric responses of polarized, aligned P(VDF-TrFE) nanofibrous scaffolds into the subcutaneous thigh region of Sprague Dawley (SD) rats (Figure 26a). To simulate the movement of SD rats, a linear motor system was employed to gently pull a leg of the SD rat under 0.5 N with 1 Hz frequency [180]. By pulling the leg of a rat gently, piezoelectric current and voltage were generated in vivo (Figure 26b,c).

**Figure 27:**
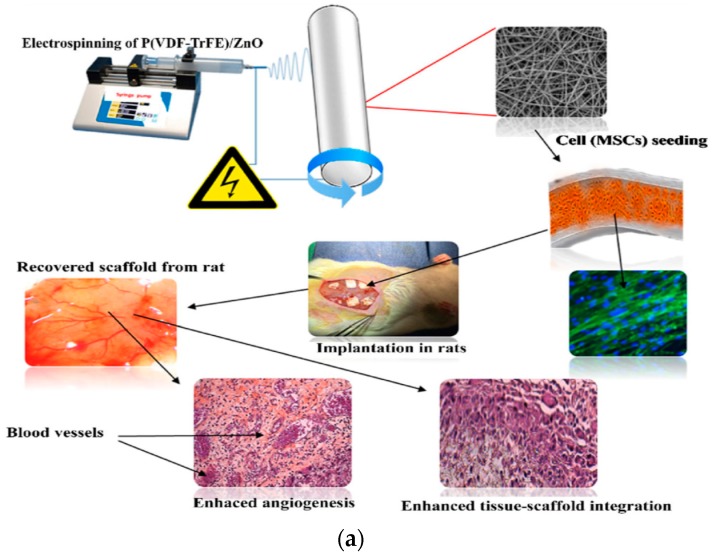

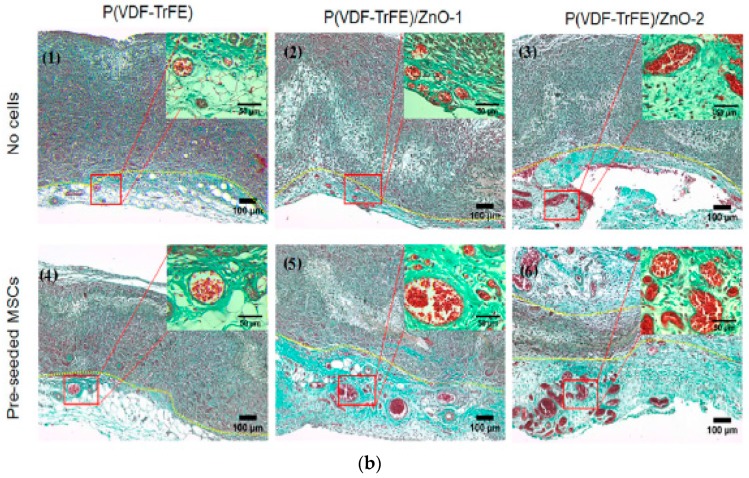
(**a**) Schematic showing the fabrication of electrospun P(VDF-TrFE) and P(VDF-TrFE)/ZnO scaffolds, hMSC seeding, and subsequent implantation into Wistar rats. (**b**) Histological examinations of fibrous scaffolds with or without pre-seeded hMSCs after implantation in rats for 7 days, and stained with Masson’s trichrome. Blood vessels developed in connective tissue adjacent to scaffolds as distinguished by yellow dashed lines, and collagen was found in all scaffolds (green). P(VDF-TrFE)/ZnO-1 and P(VDF-TrFE)/ZnO-2 contained 1 wt% and 2 wt% ZnO, respectively. Reproduced with permission from [177], published by Springer, 2017.

Augustine et al. electrospun P(VDF-TrFE) and P(VDF-TrFE)/ZnO fibrous mats; the fiber diameter of neat copolymer was 1 µm, while that of P(VDF-TrFE)/1 wt% ZnO and P(VDF-TrFE)/2 wt% ZnO nanocomposite mats was 1.05 and 1.19 µm, respectively [177]. Those fibrous mats with or without hMSCs were subcutaneously implanted into abdominal region of Wistar rats (Figure 27a). Extensive collagen fiber networks were observed in all fibrous scaffolds after implantation for 7 days. Moreover, newly formed blood vessels were readily seen in the nanocomposite scaffolds with 1 wt% and 2 wt% ZnO nanoparticles. This was further increased by pre-seeding with hMSCs in the nanocomposite scaffolds prior to implantation (Figure 27b). Thus, ZnO nanoparticles of the nanocomposite scaffolds promoted angiogenesis and favored integration of the scaffolds into the surrounding tissue [177]. In this respect, reduced neovascularization of conventional nanofibrous scaffolds can be tackled by using piezoelectric P(VDF-TrFE)/ZnO fibrous scaffolds.

### 4.2. Neural Tissue Engineering

As is widely recognized, different cell types can respond in different ways to a biomaterial surface. So different tissues need different microenvironments for sufficient cell–cell interaction, cell migration, proliferation, differentiation and regeneration [181]. In particular, piezoelectric PVDF-based materials are effective sites for the attachment, growth and differentiation of neurons with the involvement of electrical activity. In the context of neural tissue engineering, aligned electrospun fibrous mats offer a distinct advantage over the scaffolds with randomly oriented fibers. This is because highly aligned fibers provide spatial guidance for neurite outgrowth and axonal elongation.

#### 4.2.1. In Vitro Model

**Figure 28:**
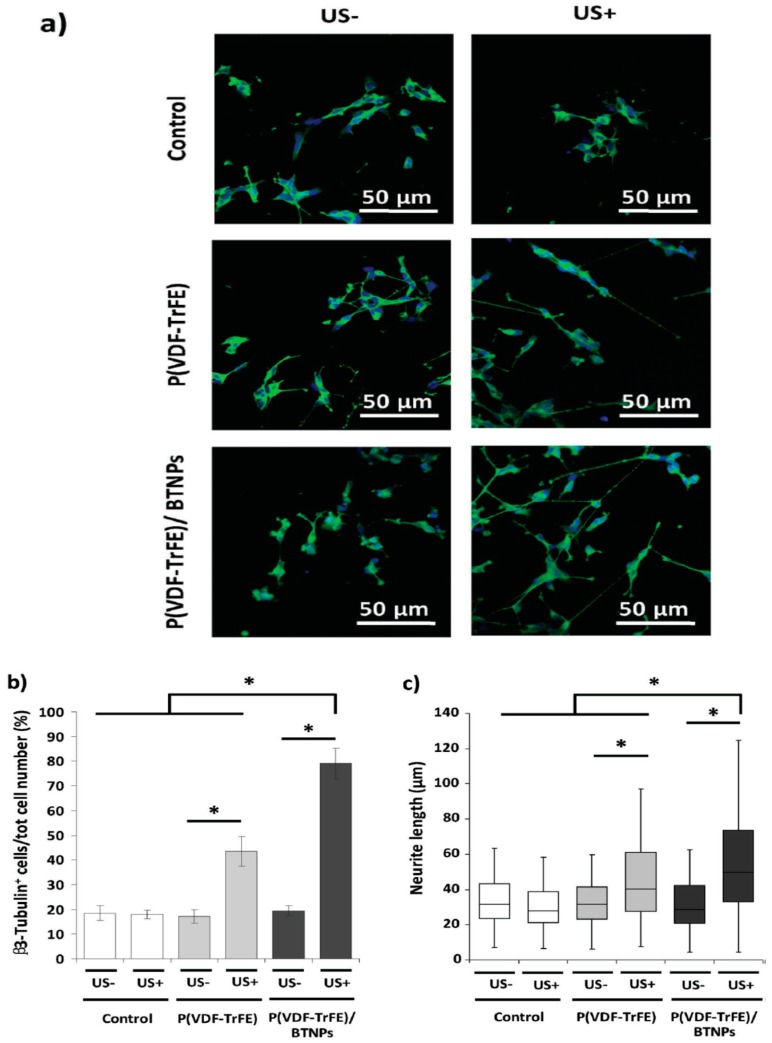
(**a**) Confocal fluorescence microscopic images of SH-SY5Y cells on solvent-cast P(VDF-TrFE) and P(VDF-TrFE)/BTNP films, as well as Ibidi (control) at the end of differentiation period of 6 days. SH-SY5Y cells were treated with or without ultrasound stimulation (US+ or US−). β3-tubulin was stained in green, nuclei in blue. (**b**) Percentages of β3-tubulin positive cells. (**c**) Neurite lengths are expressed as median values ± confidence interval at 95%. * *p* < 0.05. Reproduced with permission from [182], published by Wiley-VCH, 2016.

Genchi et al. added 60 wt% barium titanate nanoparticles (BTNPs) to solvent-cast P(VDF-TrFE) film for improving its piezoelectric properties. The BTNPs addition further enhanced the β-phase content of P(VDF-TrFE) from 30% to 50%. As a result, P(VDF-TrFE)/BTNP film promoted the viability and differentiation of human neuroblastoma cells (SH-SY5Y). Furthermore, ultrasound stimulation (US) was used to promote the adhesion and differentiation of SH-SY5Y cells cultivated on the P(VDF-TrFE) and P(VDF-TrFE)/BTNP specimens (Figure 28a,b) [182]. From Figure 28b, β3-tubulin was used as a marker for the differentiation of SH-SY5Y cells. The percentage of β3-tubulin positive cells increased markedly after US treatment, especially for the P(VDF-TrFE)/BTNP film. Ultrasound stimulation also increased neurite length of SH-SY5Y cells cultured on both P(VDF-TrFE) and P(VDF-TrFE)/BTNP films as a result of the activation of calcium channels (Figure 28c). Similarly, Hoop et al. demonstrated that ultrasonic stimulation of PC12 neuronal cells cultured on poled β-PVDF membrane activates their calcium channels, thus increasing neurite length greatly [46].

##### Fibrous Scaffolds for Neural Tissue Engineering

As mentioned, highly aligned electrospun fibers provide spatial guidance for neurite outgrowth and axonal elongation. Accordingly, neurite migration, attachment, proliferation and differentiation on aligned nanofibers are directed along the nanofiber orientation [183,184]. Moreover, electrical stimulation has been found to be particularly useful for neurite growth. Corey et al. demonstrated that neurons from the primary rat dorsal root ganglia cultured on electrospun poly l-lactide (PLLA) fibrous mats with highly aligned fibers were 20% longer than the neurites on random fibers [185]. Koppes et al. investigated neurite outgrowth on laminin-coated PLLA films and electrospun microfibers with or without electrical stimulation. The electrical stimulation increased neurite outgrowth by 32% on the films or fibers when compared to unstimulated films. In addition, neurite extension increased by 74% on the aligned fibers compared to the control film specimen [186]. PLLA also exhibits piezoelectric effect with a smaller piezoelectric constant; the shear piezoelectric constant (*d*_14_) of uniaxially stretched PLLA film is about 6–10 pC/N [187].

**Figure 29:**
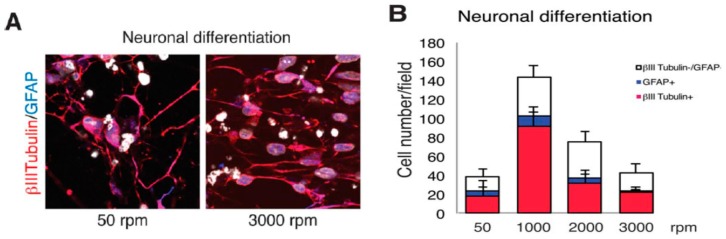
(**A**) Immunofluorescent staining for β3-tubulin (red) and GFAP (blue), and (**B**) mean percentage of cells expressing β3-tubulin and GFAP upon NSCs differentiation. n = 3–5. Reproduced with permission from [15], published by Wiley, 2017.

**Figure 30:**
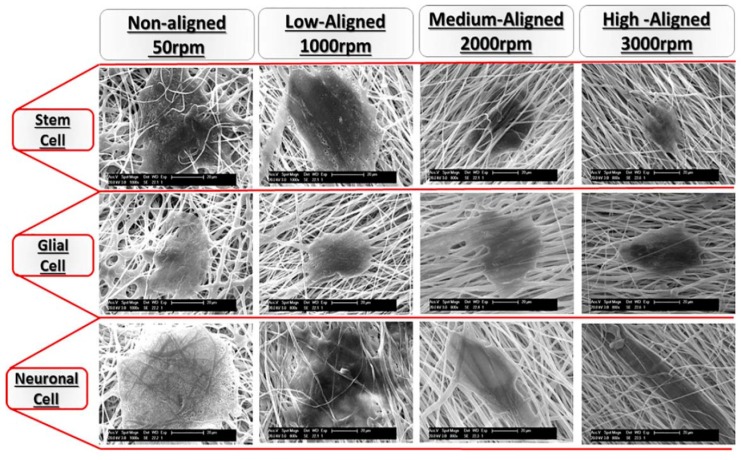
Scanning electron micrographs showing the morphologies of stem cell, neuronal cell, and glial cell cultivated on electrospun PVDF scaffolds with different fiber orientations. Reproduced with permission from [15], published by Wiley, 2017.

Lins et al. fabricated PVDF fibrous mats by rotating the drum collector for achieving non-aligned (50 rpm), low-aligned (1000 rpm), medium-aligned (2000 rpm) and high-aligned (3000 rpm) fibers. The effect of PVDF fiber alignment on the cellular responses of monkey neural stem cells (NSCs) was studied [15]. NSCs are multipotent stem cells capable of differentiating into all types of neural lineage such as neuronal and glial cells. For immunofluorescent microscopy examinations, cells were stained with β3-tubulin and glial fibrillary acidic protein (GFAP), i.e., an intermediate protein expressed in glial cell (Figure 29a). For low-aligned (1000 rpm) scaffold, the highest number of cells (143.5 cells per field) was observed in which nearly 64% of the cells expressed β3-tubulin and 8% expressed GFAP (Figure 29b). The average number of cells expressing β3-tubulin was smaller in other PVDF scaffolds. Thus, low-aligned PVDF scaffold favored the differentiation of NSCs. Figure 30 showed the SEM images of stem cell, neuronal cell, and glial cell cultured on the PVDF mats with different fiber orientations. Stem and glial cells showed irregular features, and neuronal cells had elongated morphology. The neurons were oriented parallel to the fiber direction of medium- and high-aligned mats, while they oriented randomly on low-aligned and nonaligned fibrous scaffolds.

**Figure 31:**
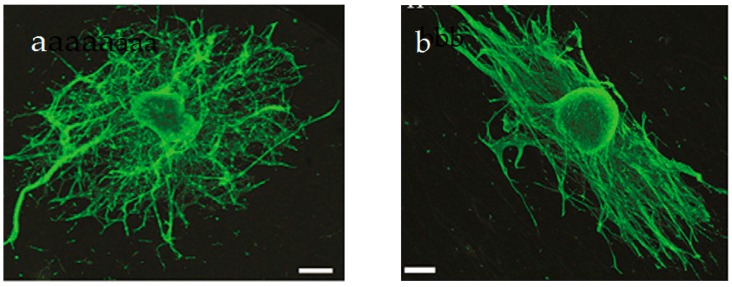

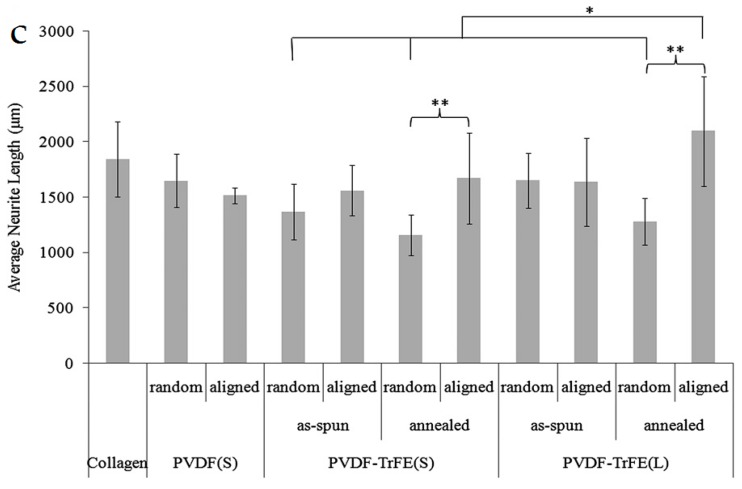
Confocal fluorescent images of DRG stained with phalloidin (actin) on micron-sized, annealed (**a**) random and (**b**) aligned P(VDF-TrFE) mats. Scale bar: 300 µm. (**c**) Average neurite length of DRG neurons cultured on micron-sized (L) and nano-sized (S) P(VDF-TrFE) mats with random and aligned fibers under as-spun and annealed conditions. Mean neurite length cultured on nano-sized (S) PVDF mats with random and aligned fibers, and collagen control are also shown for comparison. * and ** denote statistically significant difference between the sample groups; *p* < 0.05. Reproduced with permission from [6], published by Elsevier, 2011.

Arinreh and coworkers electrospun PVDF and P(VDF-TrFE) fibrous mats having both random and aligned fibers with mean diameters of micron (3.32 µm) and nanometer (750 nm) dimensions [6,188]. The random fibers were collected on a grounded metal plate, and aligned fibers were deposited on a rotating drum. The as-spun scaffolds were annealed at 135 °C for 96 h and quenched with ice water. The porosity and pore size of the micron-sized, as-spun mats were 58.08% and 4.8 µm, while those of annealed counterparts were 79.63% and 8.0 µm, respectively. The porosity and pore size of the nano-sized, as-spun mats were 43.92% and 1.7 µm, while those of the annealed counterparts were 67.55% and 1.5 µm, respectively [6]. For a given fiber size, annealed and aligned mats had higher elastic modulus and degree of crystallinity than the random and as-spun counterparts, leading to annealed mats with higher tensile strength [188]. In general, elastic modulus and tensile strength of polymers increased with increasing the degree of crystallinity [189,190,191]. Rat dorsal root ganglia (DRG) neurons were cultured on all fibrous mats for 4 days. Annealed and aligned P(VDF-TrFE) mats with micron-sized fibers showed the largest neurite extension in comparison with random mats (Figure 31a–c). This was ascribed to annealing treatment increased the β-phase content and piezoelectricity of P(VDF-TrFE) mats. Furthermore, neurite length on micron-sized, aligned and annealed mats was slightly longer than that of nano-sized counterparts. From Figure 31a, neurons with sizes of about 2000 µm including the cell body and dendrites favored micron-sized, annealed and aligned fibers for their attachment and outgrowth. Such fibrous mats exhibited the highest porosity and pore size values of 79.63% and 8.0 µm. In another study, human neural stem/progenitor cells cultured on annealed and aligned P(VDF-TrFE) mats with micron-sized fibers differentiated into neuron-liked cells as revealed by positive β3-thenubulin staining [188]. So those fibrous mats show potential applications for nerve regeneration, because in vivo piezoelectric activity in these fibers can be activated by the bulk deformations associated with cerebrospinal fluid circulation. In this respect, piezoelectric P(VDF-TrFE) mats with enhanced human stem cell differentiation are effective biomaterials for nerve tissue repair or replacement of damaged nerve cells due to the injury or disease.

**Figure 32:**
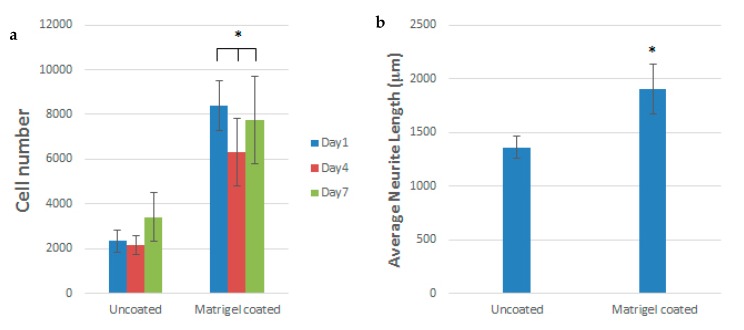
(**a**) SC number on aligned PVDF-TrFE fibrous scaffolds with or without Matrigel coating. Cell number on Matrigel coated scaffolds was significantly higher than on uncoated scaffolds at all time points (* *p* < 0.05). (**b**) Average neurite extension of DRGs cultured on uncoated and Matrigel coated PVDF-TrFE scaffolds. Neurite extension on Matrigel coated scaffolds was significantly higher than uncoated scaffolds (* *p* < 0.05). Reproduced from [194], published by Frontiers, 2018.

From the literature, aligned electrospun PCL fibers can enhance Schwann cell maturation [192]. Those fibers can mimic the natural ECM of the spinal cord, providing an ideal microenvironment at the injury site to facilitate neural repair. Schwann cells (SCs) are the glial cells of the peripheral nervous system (PNS), and cover the surface of axons of motor/sensory neurons to produce a myelin sheath and enhance nerve repair/regeneration. During injury, SCs remove damaged axons/myelin debris, promote axonal regrowth, and remyelinate axons [193]. Apparently, aligned PVDF-based scaffolds with piezoelectricity are effective biomaterials to promote Schwan cell maturation and nerve regeneration in the PNS.Very recently, Arinzeh and coworkers electrospun aligned P(VDF-TrFE) mats and then cultured SCs and DRG explants on those scaffolds [194,195]. They reported that aligned P(VDF-TrFE) mats enhance SCs growth and neurite extension, especially for Matrigel coated scaffolds (Figure 32a,b). Matrigel contains multiple extracellular matrix proteins and bioactive components extracted from mouse tumor cells [195]. Growth factors of different types are frequently tested for its influence on peripheral nerve regeneration. Therefore, aligned P(VDF-TrFE) scaffolds loaded with Matrigel are beneficial to treat peripheral nerve injury. 

#### 4.2.2. In Vivo Models for Neural Tissue Engineering

Peripheral nerve injury due to trauma remains a big challenge for the researchers nowadays. Autologous nerve grafts are typically used by medical doctors to treat the nerve damage. The use of autologous nerve grafts has some drawbacks, including donor site morbidity, limited availability, mismatch of nerves and complicated surgeries. Accordingly, synthetic nerve guide conduits have been developed to facilitate axonal growth and nerve regeneration [196]. The inclusion of electrospun PCL fibers into nerve guide conduits generally supports axonal regeneration in vivo [197]. 

Very scarce in vivo models have been conducted on the neural tissue responses to the PVDF-based scaffolds. Aebischer et al. fabricated piezoelectric PVDF nerve guidance channels. Nerves regenerated in poled PVDF channels had a higher number of myelinated axons than those regenerated in unpoled channels [198]. Very recently, Arinzeh and coworkers assessed the potential use of PVDF-TrFE conduits with SCs for spinal cord repair in vivo. In their study, the spinal cords of adult rats were completely transected. The conduits, with random or aligned fibrous inner walls, were transplanted into transected rat spinal cords for 3 weeks. The conduits with aligned fibers promoted greater axon regeneration over random fibers [199]. 

## 5. Major Challenges

Remarkable achievements have been made in recent years in the development of electrospun fibrous PVDF-based scaffolds with piezoelectric effects for bone and nerve tissue engineering. Those nanofibrous mats have a very large volume to surface ratio, thus favoring adhesion, proliferation and differentiation of osteoblasts, hMScs and neuronal cells. However, electrospun fibrous scaffolds also have some drawbacks, including small pore size, limited mat thickness, reduced neovascularization, etc. For instance, the as-spun P(VDF-TrFE) mats have a very small pore size of 1.7 µm [6]. As the pore sizes of fibrous mats are much smaller than the dimensions of individual cells, thus bone or nerve cells can only attach and proliferate on the mat surfaces. This leads to the formation of a single cell layer on the scaffold surfaces. In this respect, researchers have spent much effort developing electrospun scaffolds with larger pore sizes and porosities for cell infiltration and ingrowth. These include cryogenic electrospinning, electrospinning with salt leaching, and electrospinng with gas foaming [83,200,201].

In cryogenic electrospinning, the collector is immersed in liquid nitrogen such that ice crystals are formed in the polymer fibers. The ice particles increase the distance between the fibers acting as void spacers during the fiber deposition. They are subsequently removed by sublimation, leaving void spaces behind. This technique is based on thermally induced phase separation (TIPS) approach for inducing polymer-rich and polymer-poor regions in the fibers. The resulting porosities can reach up to 99.5% [202,203]. The salt leaching strategy involves the introduction of salt particles to the Taylor cone during electrospinning, and the particles are removed by leaching in a water bath after fiber deposition [204]. Despite the increase in the pore sizes of fibrous mats by these techniques, conventional electrospinning process can only generate two-dimensional (2D) fibrous mats with irregular pore size network due to the whipping instability. As is widely recognized, 3D electrospun fibrous scaffolds, which can mimic the natural tissue structures, are considered to be of clinical importance. This motivates the researchers to develop a near-field electrospinning (NFES) to deposit well-aligned fibers in a short distance between the needle and collector (e.g., few millimeters) to suppress bending instability [205,206]. Thus, NFES can produce complex 3D fibrous scaffolds with desirable patterns and geometries because it allows a precise control of the fiber deposition in a direct writing (DW) mode. Designed patterns can be made by monitoring the translation of the collector, and the resulting fibers are stacked to form 3D scaffold [207]. Limited studies have been conducted on the PVDF-based mats prepared by NFES. Liu et al. employed NFES to prepare PVDF/MWCNT mats with enhanced piezoelectricity [208]. Very recently, Lee et al. employed NFES to form well-aligned, 3D-PVDF fibrous mats for sensor applications [209]. Till to present, there exists no literature reports relating in vitro and in vivo behaviors of 3D fibrous scaffolds prepared by NFES. 

The main drawback of the solution-based electrospinning for biomedical applications is the use of organic solvents for dissolving polymers and for evaporating polymer jets to form fibers. In particular, DMF and DMAc solvents used for fabricating PVDF-based fibers are highly toxic and harmful to human cells [210]. Thus, melt-electrospinning can be used to fabricate fibrous mats without using toxic solvents. However, coarse fibers in the tens to hundreds of micrometers are produced in melt-electrospinning due to the high melt viscosity and low melt conductivity [155]. The low surface area to volume ratio of coarse fibers can reduce cell attachment on their surfaces. In general, the melt fluid flow with a straight flight path can be tailored to create 3D scaffolds with controlled porosity through a direct writing process, terming as the ‘melt electrospinning writing’ (MEW) [211]. MEW has been used only to create 3D PCL scaffolds due to the low melting temperature of PCL (i.e., 60 °C) [157,211,212]. By optimizing the processing parameters of PCL, direct-write PCL scaffolds with fine filaments of 817 ± 165 nm can be produced. As mentioned, non-polar α-phase is the dominant crystalline structure found in melt-electrospun PVDF fibers [160]. Therefore, great effort is required to solve the technical issues for forming melt-electrospun 3D PVDF fibrous mats containing electroactive β-phase for tissue engineering applications. 

Most literature studies have been confined to the use of 2D PVDF-based fibrous mats for cell cultivation in vitro. A systematic investigation on the cellular response to electrospun PVDF-based scaffolds in vivo is lacking, especially for the bone and neural defects. At present, only a few studies have been conducted on in vivo animal models of electrospun PVDF-based scaffolds and conduits [39,177,179,199]. For bone tissue engineering, an in-depth study and comprehensive understanding of animal models treated with the PVDF-based fibrous scaffolds is also lacking. The literature relating neuronal cell responses to the PVDF-based fibrous scaffolds in vivo is scarce.

## 6. Future Direction

For the successful implementation of electrospun PVDF-based scaffolds and conduits for bone and neural tissue engineering, bacterial infection of those scaffolds will become an issue and cannot be ignored. Surgical procedures involving scaffolds and implants are complicated by bacterial infection. Bacterial infection often leads to the morbidity and mortality of patients globally. Device-related infection is resulted from the bacterial adhesion, colonization, and biofilm formation. This is mostly caused by gram-positive *Staphylococcus aureus* (*S. aureus*). Healing of bone defects with fibrous scaffolds can be complicated by the presence of *S. aureus*, especially methicillin resistant *Staphylococcus aureus* (MRSA) [213]. Electrospun polymer scaffolds with a large surface/volume ratio can load antibiotics such as vancomycin and gentamicin in preventing infection of the bone/joint tissue and implant biofilm formation [214,215]. To prevent bacteria from developing drug resistance, we can load silver nanoparticles into electrospun PVDF-based scaffolds having no bactericidal activity to form antibacterial nanocomposites. Silver nanoparticles (AgNPs) are known to resist a wide variety of bacteria strains including gram-positive S. aureus, MRSA, and gram-negative Pseudomonas aeruginosa and Escherichia coli [51,216]. However, AgNPs can induce cytotoxicity on human neural cells and fibroblasts in a dose-dependent manner [217,218]. So AgNPs behave like a double-edged sword, having bactericidal activity, but also cytotoxicity on some human cells. Therefore, it is necessary to study the effects of AgNPs additions on the bactericidal activity, biocompatibility and electroactive β-phase formation on electrospun PVDF and P(VDF-TrFE) fibrous mats in detail. 

As mentioned above, very scarce information is available in the literature on in vivo animal studies of piezoelectric PVDF-based scaffolds for bone and neural tissue engineering. Those studies were typically carried out on mouse models for assessing bone regeneration and spinal cord repair using PVDF-based scaffolds [179,199]. However, mouse models have some drawbacks, because they are not helpful for long-term investigations in which multiple biopsies or blood samples are required. These arise from short life expectancy, and relatively small tissue and blood sample volumes of mice when compared with larger animal models such as goats and pigs [219]. In this context, more in vivo animal studies using both the mouse and large animal models are needed in the near future to investigate the biocompatibility of electrospun PVDF-based scaffolds, and an inhibiting effect of bacterial biofilm on wound healing using PVDF-based scaffolds containing AgNPs nanofillers.

## 7. Conclusions

Electrospinning merges wet chemical solution processing, electric field poling and stretching into a single step procedure for forming piezoelectric β-PVDF fibers. The incorporation of TrFE monomer into PVDF can stabilize the β-phase without mechanical stretching or electrical poling. However, mechanical stretching or electrical poling can further enhance β-phase in the P(VDF-TrFE). Piezoelectric PVDF and P(VDF-TrFE) fibrous mats find attractive applications for bone and neural tissue engineering. Bone is a natural nanocomposite consisting of collagen fibrils and nHA. Minute mechanical deformation of collagen fibrils in human bones can induce electrical potential that is crucial for bone healing and regeneration. Accordingly, piezoelectric PVDF and P(VDF-TrFE) fibrous mats promote osteoblastic adhesion, proliferation and differentiation on their surfaces in vitro. The hMSCs cultivated on PVDF scaffolds under electrical stimulation exhibit high ALP activity and biomineralization. Furthermore, aligned PVDF and P(VDF-TrFE) fibrous mats can direct neurite growth, promote neural stem cell differentiation and support the growth of Schwann cells. Therefore, those fibrous mats can be used as potential biomaterials for making nerve guidance conduits for treating peripheral nerve damage. 

The main drawbacks of the electrospinning process for making piezoelectric PVDF-based scaffolds for tissue engineering applications are the formation of very small pores and the use of organic solvents. Conventional two-dimensional PVDF-based scaffolds with very small pores prevent the infiltration of bone and neuronal cells into the scaffolds. Consequently, those cells can only attach and proliferate on the scaffold surfaces, forming a single cell layer with limited cell infiltration. Near-field electrospinning designed for sensor applications can create 3D PVDF fibrous mats for tissue engineering [209]. However, there are no literature articles reporting in vitro and in vivo behaviors of 3D fibrous PVDF-based scaffolds prepared by NFES. The main limitation of NFES for tissue engineering is the use of organic solvents. The solvents typical used for fabricating PVDF fibers are DMF and DMAc, which are toxic to human cells. Melt electrospinning with a direct writing process can create 3D scaffolds with controlled porosity without using organic solvents. However, MEW process is currently confined to make PCL fibrous mats due to the ease of processing as PCL has a low melting temperature. Much efforts are needed by the researchers to develop solvent free, electrospun 3D PVDF-based scaffolds with interconnecting pore networks for bone and neural tissue engineering applications. 

## Figures and Tables

**Table 1:** Effect of β-phase content on piezoelectric coefficient of electrospun PVDF and its nanocomposite mats.

Materials	β-Phase Content	Fiber Diameter, nm	*d*_33_ Value, pm/V	*d*_33_ Value, pC/N	Ref.
PVDF random fibers	NA	1410	—	24.90	[39]
PVDF random fibers	79 ± 3	155 ± 17	—	16.8 ± 1.4	[134]
PVDF aligned fibers	88 ± 1	118 ± 23	—	27.4 ± 1.5	[134]
PVDF/MWCNT aligned fibers	89 ± 2	116 ± 21	31.3 ± 2.1	31.3 ± 2.1	[134]
PVDF random fibers	78	1500	30	—	[147]
PVDF/1% MWCNT random fibers	84	300	35	—	[147]
PVDF/1% (MWCNT-AgNP) random fibers	89	800	54	—	[147]
PVDF/1% GO random fibers	70	623	40	—	[148]
PVDF/1% CGO random fibers	79	622	46	—	[148]
PVDF/1% FGO random fibers	89	619	63	—	[148]
Human bones	—	—	—	7–8	[26]

NA: Not available.
